# VI-VS: calibrated identification of feature dependencies in single-cell multiomics

**DOI:** 10.1186/s13059-024-03419-z

**Published:** 2024-11-15

**Authors:** Pierre Boyeau, Stephen Bates, Can Ergen, Michael I. Jordan, Nir Yosef

**Affiliations:** 1 Department of Electrical Engineering and Computer Sciences, University of California, Berkeley, USA; 2 Department of Statistics, University of California, Berkeley, USA; 3 Center for Computational Biology, University of California, Berkeley, USA; 4 Department of Systems Immunology, Weizmann Institute of Science, Rehovot, Israel; 5 Inria, Paris, France; 6 Department of Electrical Engineering and Computer Science, Massachusetts Institute of Technology, Cambridge, USA

## Abstract

Unveiling functional relationships between various molecular cell phenotypes from data using machine learning models is a key promise of multiomics. Existing methods either use flexible but hard-to-interpret models or simpler, misspecified models. VI-VS (Variational Inference for Variable Selection) balances flexibility and interpretability to identify relevant feature relationships in multiomic data. It uses deep generative models to identify conditionally dependent features, with false discovery rate control. VI-VS is available as an open-source Python package, providing a robust solution to identify features more likely representing genuine causal relationships.

**Supplementary Information:**

The online version contains supplementary material available at 10.1186/s13059-024-03419-z.

## Background

Single-cell transcriptomics offers an unprecedented opportunity for probing the function of individual cells and for characterizing the cellular composition of entire samples, thus shedding new light on processes in immunity, development, and pathogenesis of various diseases [1–4]. The emergence of spatial and multiomic technologies further adds the ability to simultaneously profile the surface proteome, epigenome, or location of each cell, on top of its transcriptome. In addition to providing a more comprehensive view of each cell, these technologies open the way for a better understanding of the interplay between molecular or cellular properties. For instance, assessing the dependency between protein abundance on the cell surface and the expression of genes can help identify signaling cascades that help propagate extracellular cues and induce a transcriptional response [5]. Identifying associations between gene expression and the cell’s epigenetic landscape [2] may further help with our understanding of how gene expression is regulated. In spatial transcriptomics, an examination of gene expression patterns across tissue localizations may reveal how the microenvironment affects the function of its residing cells [6]. All of these opportunities require statistical procedures to help detect the most relevant relationships between the observed molecular or cellular features (genes, proteins, chromatin regions, cellularity of the microenvironment, and more).

**Fig. 1:**
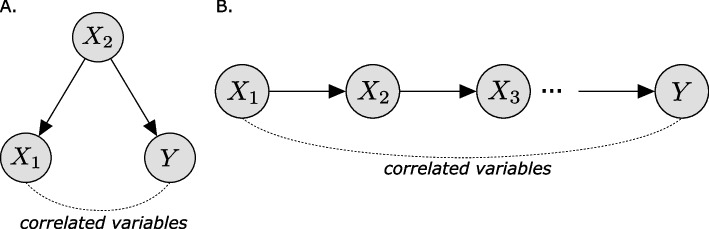
Correlated variables may be functionally unrelated. Here, $X_1, X_2, Y$ are random variables characterizing the expression of three genes. **A** $X_2$ is directly and causally linked to *Y* and $X_1$. Here, $X_1$ and *Y* might be highly correlated, but $X_1$ does not causally affect *Y*. **B** $X_1$ is directly and causally linked to $X_2$, and similarly, $X_2$ is connected to *Y*. Here, $X_1$ and *Y* might be highly correlated, but their association is indirect

In single-cell genomics and bulk settings, efforts to detect relationships between such features fall into two broad categories. The simplest methods identify *marginal* associations, which quantify statistical dependencies between pairs of features without considering the other observed features. While these were broadly used for studying gene co-expression networks [7–10], marginal associations suffer from key limitations for single-cell genomics. Practically any technology in this field is impacted by technical factors such as batch effects or variation in sequencing depth as well as “nuisance” biological factors that are less relevant to the question in hand, e.g., the cell cycle. These factors may inflate marginal correlations, resulting in associations that do not carry the intended biological meaning [11]. More fundamentally, a marginal correlation between two variables in any arbitrary system does not imply causation [12, 13]. For instance, two genes that are regulated by a common set of transcription factors can be highly correlated without being functionally related (Fig. 1A). Even when they are functionally related, marginal dependencies may not inform on the proximity of this relationship when two highly correlated genes are indirectly linked through a series of mediator genes (Fig. 1B). Marginal approaches hence tend to detect many spurious or indirect associations, which requires further filtering to identify the most relevant relationships [7, 14, 15].

*Conditional associations* are a second category of relationships that address these issues by accounting for the overall dependency structure of the data when assessing the dependency between a pair of variables [16–20]. Specifically, detecting conditional dependencies between a response variable *Y* and individual features in a feature matrix *X* often starts by learning a predictor function, $f(X)\approx Y$, which is then scrutinized to identify variables in *X* that are most associated with *Y*. The simplest example for this approach is the generalized linear model [21, 21–23], in which learned regression coefficients are used to quantify conditional associations. While limited to linear relationships and simple noise models, linear approaches are relatively scalable. In some cases, these models come with statistical guarantees for the inferred coefficients and are thus easily interpretable. Nonlinear predictors [16, 17] have also been introduced to capture more complex relationships, with tree ensembles being the most prevalent approach. Ensemble approaches have been demonstrated to reach state-of-the-art performance in a variety of tasks such as inference of regulatory interactions between genes [24]. Conditional dependencies provide a more stringent notion of association than marginal dependencies and are more likely to reflect causal relationships. Indeed, pairwise dependencies that persist after conditioning on all other variables imply causal relationships in cases where the causal direction is known, there are no unobserved causal variables, and there is no feedback loops [25]. As such, conditional dependencies are a promising avenue for uncovering relevant interactions in single-cell multiomic data.

In practice, however, algorithms for identifying conditional relationships often need to compromise on (i) *scalability*, e.g., requiring heavy pre-processing to ensure that inference can be completed in a reasonable timeframe [24], (ii) *modeling assumptions*, using often mis-specified view of the underlying process, e.g., with simplified noise models or by assuming linear relationships between variables [26], and, importantly, (iii) *interpretability and calibration*, by relying on heuristics to evaluate which of the interactions under consideration are indeed relevant [27–29]. Given these challenges, analyzing dependencies in single multiomics, where millions of measurements (possibly from different batches or studies) are available, requires the use of scalable and rigorous statistical methods. These methods should be able to handle count data distributions, account for technical and biological noise and bias, and allow for nonlinear relationships between variables.

To address these three challenges, we introduce VI-VS (Variational Inference for Variable Selection), a general framework for conditional independence testing with multiomic data. VI-VS is based on the conditional randomization test (CRT) [30], which quantifies the credibility of pairwise interactions by measuring the effect of exchanging observed features with synthetic ones. We demonstrate and theoretically prove that our procedure provides a calibrated estimation of the false discovery rate. This is achieved without making any assumptions about the distribution of the response variable *Y* or the nature of its interactions with the features in *X*, such as linearity. VI-VS harnesses the distributional expressivity of latent variable models, allowing for a variety of noise models for *X*, including count distributions commonly used in single-cell genomics. Finally, VI-VS relies on deep neural networks for testing, allowing it to scale to large single-cell genomic datasets as well as capture complex nonlinear relationships between variables.

In the following, we demonstrate the accuracy and calibration of VI-VS with several simulation and multi-ome case studies. We also showcase that our procedure provides a theoretically grounded “wrapper” framework that can take existing algorithms for detecting pairwise relationships and use them to output calibrated decisions. We demonstrate this using the popular GENIE-3 algorithm for inference of regulatory networks.

## Results

### Variational Inference for Variable Selection

VI-VS is a conditional independence testing framework for single cell genomics. We observe, for multiple cells, *G* features $X_1, \dots X_G$, e.g., genes, as well as a response variable *Y*, e.g., measured protein expression levels, or another type of cellular property. This section omits the confounding factor adjustment for simplicity. We refer to the “Methods” section for a detailed description of the full algorithm in the presence of confounding factors.

Conditional independence tests identify features for which the null hypothesis of conditional independence $\mathcal {H}_{0, g} : X_g {\perp\mkern-10mu\perp} Y \mid X_{-g}$ can be rejected. Here, $X_{-g}$ denotes the set of features $X_1, \dots , X_G$ excluding $X_g$. Conditionally dependent features, for which the null can be rejected, are associated with the response variable in a way that cannot be explained by the other features. This allows for the identification of features that have a distinct and significant association with the response variable and are less likely to be the consequence of spurious correlations among features.

**Fig. 2:**
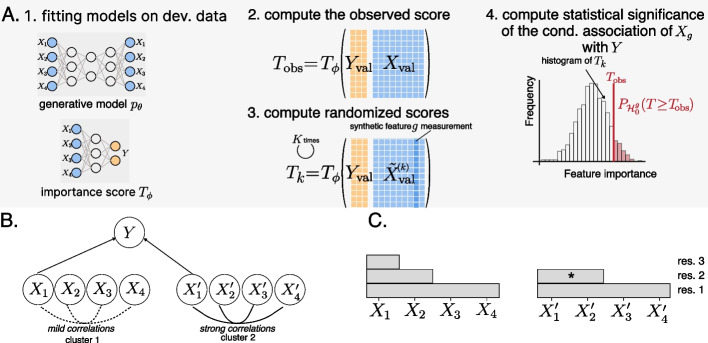
**A** VI-VS overview. VI-VS identifies conditional dependencies between molecular features *X*, e.g., genes, and a response variable *Y* of cell properties, e.g., protein expression levels, in single-cell genomics. (1) We first randomly split the observed data into a *validation* and a *development* set. On the development set, we fit a generative model $p_\theta$ and importance score $T_\phi$, which is a scalar-valued function taking *Y* and *X* as inputs on the development set. In a simple case, $T_\phi$ may correspond to the prediction error of the ordinary least squares of *Y* on *X*. Here, $\theta$ and $\phi$ denote the parameters of these models, learned on the development set. (2) We compute the importance score of the observed data on the validation set. (3) In parallel, we sample *K* *synthetic* feature samples for gene *g* using the trained generative model. We then compute synthetic importance scores, computed based on *Y* and on the modified feature matrix *X* where the *g*th column was replaced by the synthetic samples. (4) We compare the observed importance score to the distribution of synthetic importance scores to compute a *p*-value. **B** Power limitation of conditional approaches. Features $X_1, X_2, X_3, X_4$ are mildly correlated and form a first cluster. Features $X_1', X_2', X_3', X_4'$ are strongly correlated and form a second cluster. If the target response causally depends on $X_1$ and $X_1'$, then a conditional independence test may fail to detect $X'_1$ due to its strong correlations with features of the same cluster. **C** Illustrative example of multi-resolution testing on **B** in the case where conditional dependencies at assessed at three resolutions (res. 3 being the finest at the feature level). VI-VS can detect groups of features that are conditionally dependent on the response variable, even if no individual gene can be identified as conditionally dependent, as well as individual features, if the sample size allows. For instance, VI-VS could detect, in additional to individual feature $X_1$, that group $\{X'_1, X'_2\}$ (marked as a star in the figure) is conditionally dependent on the response without being able which of the two features is responsible for the association

VI-VS compares observed data statistics with statistics of synthetic data generated under the null hypothesis of conditional independence (Fig. 2A). It requires two core components: (i) a way to generate synthetic data and (ii) a procedure to compare observed with synthetic data. To generate synthetic data, VI-VS employs a *generative model*, more particularly a latent variable model, trained on a subset of the available data. The generative model can be seen as a simulator that generates synthetic data in the scenario where $\mathcal {H}_{0, g}$ holds. More specifically, the generative model produces *K* synthetic measurements for feature *g*, $\tilde{X}_g^{(1)}, \dots \tilde{X}_g^{(K)}$ that are consistent with $\mathcal {H}_{0, g}$. In other words, no matter if the observed $X_g$ is conditionally dependent on *Y*, the synthetic samples $\tilde{X}_g^{(k)}$ will be conditionally independent on *Y* by construction.

The next step is to compare the observed and synthetic data to assess the credibility of $\mathcal {H}_{0, g}$ via *importance scores*. Importance scores are data statistics that concisely summarize the relationship between the features and the response variable and that we can use to compare the observed and synthetic data. A simple example of an importance score is the prediction error of a regression model of *Y* given *X*, e.g., a neural network or a linear model trained on a subset of the observed data. If $\mathcal {H}_{0, g}$ were true, then the value of feature *g* should not be informative for predicting the response. In particular, replacing $X_g$ with a synthetic counterpart $\tilde{X}_g^{(k)}$ in the input of the regression model should not significantly change the prediction error. On the other hand, if $X_g$ is conditionally dependent on *Y*, then $X_g$ should be informative for prediction, and the replacement operation should lead to a significant increase in the prediction error.

Following this logic, and in the general setting, we let *T*(*X*, *Y*) denote the importance computed on the observed data, and $T(\tilde{X}^{(k)}, Y)$ as the one computed from inputs $\tilde{X}^{(k)}$, where $X_g$ was replaced by $\tilde{X}_g^{(k)}$. VI-VS compares the observed importance score to the histogram of the synthetic importance scores to compute a *p*-value for $\mathcal {H}_{0, g}$ as

$$\begin{aligned} p_g = \frac{1}{K+1} \left( 1 + \sum \limits _{k=1}^K \mathbb {I} \left( T(\tilde{X}^{(k)}, Y) \le T(X, Y) \right) \right) . \end{aligned}$$

#### Theoretical guarantees

The *p*-values computed by VI-VS are calibrated; in particular, they control the false discovery rate (FDR) at the desired level, regardless of the complexity of the relationship between the response variable and the features. The core assumption of VI-VS is that the generative model can describe the statistical relationship between features $X_1, \dots X_g$. While not described in this section, VI-VS adjusts for confounding factors, e.g., batch effects, that may affect the relationship between *X* and *Y*. We refer to the “Methods” section for a detailed description of the full algorithm.

Importantly, *any* importance score can be used: while poor importance scores will lead to low power, the FDR will remain controlled. This property has strong consequences for the method’s versatility and validity. First, the relationship between *Y* and *X* does not need to be understood or modeled properly. For instance, an importance score built on a linear model will still provide calibrated *p*-values when the relationship between *Y* and *X* is non-linear. Second, importance scores can be built from the output of existing feature selection algorithms that do not inherently provide *p*-values, allowing VI-VS to act as a meta-algorithm that makes interpretable decisions on top of these algorithms.

#### Multi-resolution approach to feature detection

In practice, conditional independence tests may not detect many features, e.g., due to limited sample sizes when some features are highly correlated. Consider a hypothetical experiment where features form two clusters and the is a function of one feature in each cluster (Fig. 2B). A conditional independence test may fail to detect either of these features if there are strong correlations within the clusters (see, for instance, [31]). VI-VS includes a multi-resolution testing procedure that identifies conditionally dependent feature groups in addition to individual features to address the power issue of conditional independence tests. This allows for the detection of feature groups for which no individual feature can be identified as conditionally dependent on the response variable, allowing to avoid missing relevant associations. Figure 2C provides a preview of the output of this approach in the illustrative example above.

#### Implementation

We implemented VI-VS in a fast and scalable fashion, parallelizing synthetic data generation across genes and samples via GPU acceleration (Additional file 1: Algorithm S1). The algorithm is implemented in Jax and is available as a Python package at https://github.com/YosefLab/VIVS.

#### Experimental setup

We used scVI [32] as the generative model for features, corresponding to gene expression, reimplemented in Jax with its default parameters. Importance scores were calculated as the prediction errors of regression models of *y* given *x*, *s*, either corresponding to a linear model or an MLP. To train these models, we randomly split the available data into a 70–30% development-validation split. Both the generative model and the importance scores were trained on the development split; *p*-values and cell scores were computed on the validation split. Note that the generative model and importance scores need only be fit once, upstream of the CRT. In cases where our experiments contained multidimensional response variable *y*, we applied VI-VS separately and in parallel to each dimension. In such cases, however, the generative model only needs to be trained once (Additional file 1: Supplement D.4).

### VI-VS provides calibrated decisions in a semi-synthetic experiment

We considered a scRNA-seq dataset of 6,855 peripheral blood mono-nuclear cells (PBMC) [33] from a healthy human donor, with 500 genes. We generated five synthetic response variables, each corresponding to the expression of a surface protein measured in the observed cells. The expectations of each response variable were calculated as a linear combination of the *squared* values of mean-centered log count per million (CPM) expression levels of 150 randomly selected genes in *X*. These simulated response variables were further corrupted by the addition of Gaussian noise. This simulation represents a case where simple linear assumptions do not hold, but there is still a relatively simple model that connects *y* to a subset of features in *X*. More details on data generation can be found in Additional file 1: Supplement A.2.

We compared VI-VS to two baselines. Ordinary least squares (OLS) is a canonical method for conditional independence testing. For OLS, we regressed the response variables *y* on *X*, under linear and Gaussian assumptions, and used a *t*-test to estimate significance of each coefficient. We also considered a simpler (marginal) independence test baseline. For each gene *g*, we regressed *y* on the expression of *g* only and used a *t*-test to estimate significance. These two baselines used all available data, i.e., both the development and validation splits, to fit the regression models, thus lending a natural advantage over the way VI-VS was fit.

**Fig. 3:**
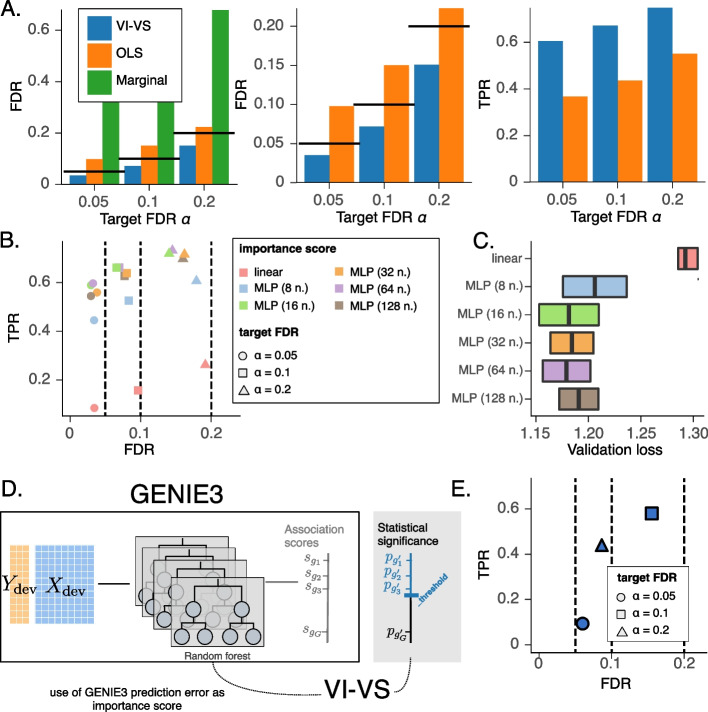
Semi-synthetic experiment. **A** Comparison of FDR control and power for conditional independence testing at the *gene* level, averaged over five random weights initializations for the models of VI-VS, and across the five surface proteins of the dataset. Here, VI-VS uses a neural network with 64 units to compute importance scores. *Left*: FDR control comparison for the CRT, ordinary least squares (OLS) under *t*-tests, and marginal independence tests. Because the marginal test did not control the FDR, it was removed from the rest of the experiments. *Center*: Zoom on the previous figure. *Right*: Associated TPR. **B** FDR-TPR curves for different importance scores averaged over five random weights initializations for the models of VI-VS and across the surface protein of the dataset. Circles, squares, and rectangles respectively represent the models’ decisions for target FDR levels of 0.05, 0.1, and 0.2. **C** Associated held-out mean squared error of the different regression models used as importance scores. **D** Use of VI-VS as a calibration tool for GENIE3. After fitting the regression tree ensemble of GENIE3, we used their prediction error as importance scores for VI-VS, allowing one to detect conditionally dependent genes with statistical significance. **E** FDR/TPR levels of VI-VS using GENIE3 reconstruction losses as importance scores. In this experiment only, for scalability reasons, we considered a total of 100 genes in the experiment. In **B** and **E**, dashed lines denote target FDR levels

We first evaluated the extent of type I error of the different algorithms (Fig. 3A). We found that the FDR estimates of the marginal independence test exceeded target levels, leading to many false positives. These false positives likely reflect indirect correlations, that is, genes that were not used to generate the synthetic response variable but strongly correlate with other genes used for data generation.

The OLS performed better but still overestimated the FDR, possibly due to the violated linearity assumptions. In addition, likelihood misspecification, i.e., invalid Gaussian assumptions on the data, can cause FDR miscalibration for OLS. As an illustration, we repeated the simulation, this time generating the response variable counts from a Poisson distribution (Additional file 1: Figure S1), in which case OLS performs worse. On the other hand, the application of VI-VS with an MLP for the importance score controlled the FDR in both these scenarios. We also evaluated the robustness of our approach to key characteristics of the simulated data, including sparsity and the number of genes in the assay, and show that our approach compares favorably to OLS, and provides consistent FDR control across all parameter settings (Additional file 1: Figure S2). Finally, we confirmed the robustness of VI-VS to the choice of development set size in Additional file 1: Figure S3, where we also suggested strategies to choose the development set size and assess calibration when necessary.

#### Increased power using more complex importance score functions

Using better importance scores can increase the power of the CRT framework (i.e., lower type II error), while still maintaining calibration of the type I error estimates (as stated by Proposition 1 in the “Methods” section and [30]). To illustrate this, we considered a range of increasingly complex model choices to compute the importance scores and repeated our simulation analysis (Fig. 3B). We found that all importance scores controlled the FDR at different levels. For instance, using linear regression with an OLS objective to compute importance scores still controlled the FDR, although this model is a poor predictor of the response variable. Conversely, higher-capacity models relying on complex MLP architectures led to increased power, indicating that more pertinent importance scores may lead to more discoveries. We also found that the models with the best held-out predictive performance also detected more true positives (Fig. 3C), providing an empirical strategy to design importance scores with VI-VS. Therefore, we advise using predictive performance as a criterion to select the importance score to use with VI-VS.

#### Using VI-VS to calibrate existing algorithms for the identification of feature interactions in single-cell genomics

Our framework can build interpretable decisions on top of existing algorithms that lack a scalable or otherwise principled way to define calibrated decision rules. An example of such a model is GENIE3, which uses an ensemble of regression trees to produce scores that quantify the importance of each gene in predicting a held-out “response” gene, thus identifying putative interactions between genes. These scores were shown to have state-of-art performance in ranking putative interactions from the most to the least relevant. They, unfortunately, do not easily inform which interactions should be considered relevant for decision-making. Consequently, we used VI-VS to construct interpretable decisions on top of GENIE3. To do so, it sufficed to plug in the GENIE3 model as importance score. Specifically, given a response variable *y*, we trained GENIE3 regression trees once using the development part of the data. We then used the respective prediction errors on the validation data as importance scores for VI-VS (Fig. 3D; Additional file 1: Supplement D.5 for details). Application to our simulated data demonstrates that this wrapper procedure provides decision rules that control the FDR at several levels (Fig. 3E), while still providing large true positive rates. The CRT framework can therefore be used to better utilize a large family of algorithms in this area, as long as they produce an estimate of interaction “strength” that considers all features in *X* serving as plugin importance score.

#### Multi-resolution testing as a way to increase power

**Fig. 4:**
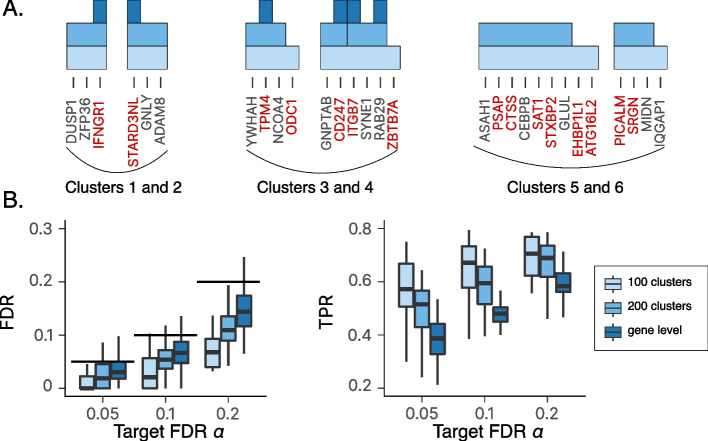
**A** Examples of gene groups identified by VI-VS in the semi-synthetic experiment. Clusters 1 and 2 show examples where all genes affecting the surface protein expression in the simulation are detected at the gene level. Clusters 3 and 4 show examples where some of the genes are not detected at the gene level but are detected at coarser resolutions. Clusters 5 and 6 show examples where none of the genes are detected at the gene level but are detected at coarser resolutions. **B** FDR (*left*) and power (*right*) for VI-VS applied at different resolutions. When testing at the group level, a group of genes was considered a true positive if it contained at least one gene that was a true positive at the gene level

The limited true positive rate in our simulation results can be explained to some extent by gene correlations that could not be resolved because of the limited size of the data. We next tested whether multiresolution could mitigate this. To this end, we applied our hierarchical procedure with different gene cluster granularities (here, 200 clusters, 300 clusters, or a per-gene analysis; Fig. 4A). We generally observed that the detections were consistent across the different resolutions, i.e., if a group of genes was identified at a given resolution, groups containing these genes were detected at coarser resolutions. Testing at multiple resolutions is useful to identify clusters of genes that were not detected at the gene level due to sample size limitations. Clusters 5 and 6 are such examples, illustrating cases where relevant genes might not be detected at the gene level, but could detected at coarser resolutions.

For a more quantitative evaluation of the merits of multi-resolution testing, we computed FDR and power, where a selected group of genes was considered true positive if it contained at least one gene that was a true positive (at the gene level) and false positive otherwise (Fig. 4B). Our results show that testing at coarser resolutions, i.e., grouping genes together, yielded more discoveries while maintaining calibrated FDR. More generally, multi-resolution testing provides more insight into the statistical relationships between genes and responses. When a coarse gene group is detected, the identification of finer-grained gene groups can be used to identify the individual genes that are responsible for the association [34]. Tying individual genes with coarser gene groups can also help identify and annotate genes with additional sources of information [35].

### VI-VS identifies causal interactions in perturb-seq data

We also considered a perturbational screen assay [36] to assess the relevance of VI-VS in identifying causal gene-gene interactions in single-cell genomics. As they can identify true causal interactions between genes, perturbational assays constitute a natural choice to benchmark the different models to produce causal candidates. To benchmark our approach, we restricted the analysis to the ten target genes with the largest number of guides. We first identified for each target gene true positive genes as differentially expressed genes using a *t*-test comparing gene expression between cells with and without guide RNA. We then fit the different models on unperturbed cells using the measured target gene expression as the response variable.

**Fig. 5:**
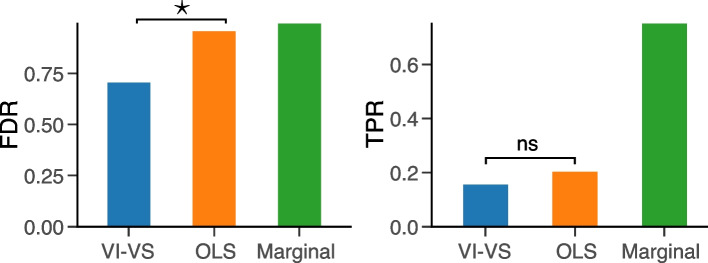
Perturb-seq experiment. *Left*: FDR and *Right*: TPR for the different models for the identification of causal interactions identified from the perturbational assay estimated from the ten target genes with the largest number of guides in the assay (target FDR level: $\alpha =0.1$). A star (*) indicates a significant difference between the metrics based on a paired t-test with a significance level of 0.05; nc indicates that the difference is not significant

Figure 5 shows the estimated FDR and TPR for the different models evaluating the match between t-test of perturbed and unperturbed cells and genes significant predictive genes estimated by the different models. As expected, the marginal approach had the highest TPR, but also the highest FDR. VI-VS obtained a significantly lower FDR than both OLS and the marginal approach (70% for VI-VS; 95% for OLS). In other words, almost a third of the associations identified by VI-VS were true positives, which is a major increase over the other models. The improved precision of VI-VS did not affect its power. VI-VS identified a similar number of true positives as OLS but from a smaller pool of detected associations (Additional file 1: Figure S4). In other words, our approach produced a smaller set of associations with high precision, which contained a similar number of true positives as the larger set of associations produced by OLS. The superiority of VI-VS over OLS for causal candidate identification was further supported by significantly higher F1 scores (Additional file 1: Figure S4).

### VI-VS identifies links between surface proteins and gene expression programs with CITE-seq

**Fig. 6:**
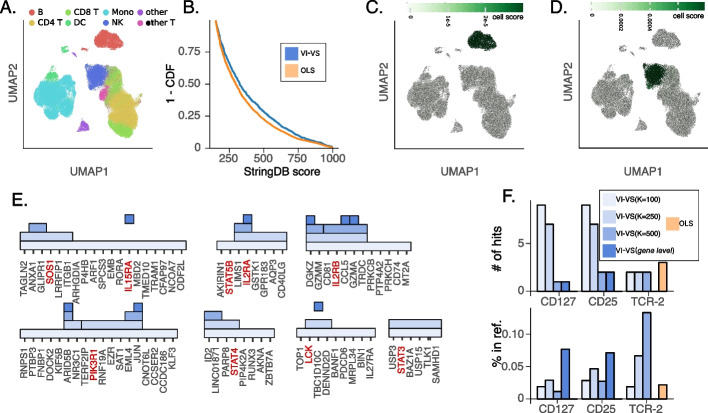
CiteSeq experiment. **A** UMAP of the dataset. **B** Distribution of stringDB scores of gene-protein discoveries made by VI-VS and least-squares (higher is better). **C**, **D** cell scores (averaged per cell-type) for the CD86-HLA and CD48-2b4 gene-protein pairs detected by VI-VS. High scores identify cells where the dependency is most expressed. **E** Visualization of VI-VS detections at several resolutions for surface protein CD25 and T cells. Each filled rectangle characterizes a gene group detected as significant by VI-VS when testing for conditional independence at several resolutions ($K \in \{100, 250, 500\}$ and at gene level). Genes in red correspond to genes contained in *Interleukin-2 Family Signaling R-HSA-451927* or *Interleukin-2 Signaling R-HSA-9020558* pathways. **F** Agreement of the predictions with the Reactome pathway database, focusing on T cells for three surface proteins. *Top*: Number of predicted genes contained in each pathway, and *bottom*: proportion of predicted genes contained in each pathway over the total number of detections. The following pathways were considered: *Interleukin-7 Signaling R-HSA-1266695* for CD127, *Interleukin-2 Family Signaling R-HSA-451927* and *Interleukin-2 Signaling R-HSA-9020558* for CD25, and *TCR Signaling R-HSA-202403* for TCR-2. For **E** and **F**, models were fit on T cells only

Next, we considered a CITE-seq dataset of PBMCs, obtained from eight healthy human donors [37]. We applied VI-VS at the single gene level, as well as the OLS baseline to a subset of this data with a total of 50,000 cells, 2000 genes, and 224 surface proteins (Fig. 6A). Notably, this dataset includes information from 13 batches, which could be accommodated by VI-VS due to the batch correction capacity built into its generative model [32]. Application of OLS to this case study (using an FDR cutoff of 10%) returned a very large number of associations for all 224 proteins (Additional file 1: Figure S5A). In contrast, VI-VS (with the same target FDR) only predicted associations for 51 of the proteins, with a smaller number of interactions per protein (52 interactions on average, compared to 140 interactions with OLS). To understand this, we first considered the set of proteins that had no associations with VI-VS. Using TotalVI [38], we estimated for each protein the percentage of cells that plausibly express it on their surface (accounting for background signal, which is often observed in protein quantification with CITE-seq). We found that those proteins with no detection by VI-VS tend to have a much lower signal, compared to the ones that have been associated with gene expression (Additional file 1: Figure S5B). Conversely, the OLS analysis identified associations for proteins that are likely not well captured or not expressed in these settings. OLS detected numerous associations (21, 41, 121, and 123 gene-protein pairs respectively) for four negative control proteins (Rat-IgG1-1, Rat-IgG2b, Rat-IgG1-2, and Rat-IgG2c) that are not expressed by human cells. In contrast, VI-VS detected none of these associations.

To further compare the validity of the associations made by VI-VS and OLS, we used StringDB [39] to evaluate the a priori support for each interaction. Specifically, we assigned StringDB’s protein-protein “combined score” to each protein-gene pair. This combined score is a composite measure that integrates the scores of protein-protein associations computed across several modalities. We first compared the distribution of these scores for predicted gene-protein interactions across all proteins (Fig. 6B). We found that the scores of the interactions predicted by VI-VS were significantly higher than the ones identified by OLS (Kolmogorov-Smirnov test, $P \le 10^{-6}$). A similar trend was observed when comparing these scores for proteins for which both methods made predictions (Kolmogorov-Smirnov test, $P \le 0.05$). This, combined with the fact that OLS detected associations for several negative control proteins, suggests that OLS is likely misspecified and may return many false positives. Conversely, VI-VS provides a more conservative and accurate way to identify biologically meaningful associations.

#### Locating protein-gene associations to the relevant cell subsets

A core feature of VI-VS is the ability to not only identify the association of genes with the response variable but also highlight the set of cells in which this interaction is more likely to be relevant. As a first example of this, we consider an association detected between MS4A1 (encoding the B cell marker CD20) and the HLA-DR receptor. Using the cell-specific importance scores from Equation 7 of the “Methods” section, we identified B cells as the most relevant cells for this association (Fig. 6C). This agrees with previous findings on the physical and functional association between CD20 and MHC-II in activated B cells [40] and the use of these two molecules as joint targets for combination therapy in lymphomas [41]. VI-VS also identified an association between the presentation of CD48 on the cell membrane and the expression of 2B4, which encodes the activating NK cell receptor CD244. The cell-specific importance scores suggest that this dependency is primarily driven by natural killer (NK) cells (Fig. 6D). This result agrees with reports on the functional association between CD48 and CD244 in NK cells, where direct binding of these molecules is important to drive the surface expression and phosphorylation of CD244 in NK cells, consequently affecting their effector function [42].

When the practitioner has prior knowledge about the cell types of interest for the analysis, it is advantageous to fit the model only on these specific cells rather than the entire dataset. This choice reduces the computational cost of the algorithm and yields clean type-specific associations, eliminating the need for post-processing based on cell-specific importance scores. To illustrate how VI-VS can unveil biologically relevant associations at multiple resolutions, we searched for associations between genes and proteins in T cells specified before testing. We focused on the CD25 surface protein (IL2RA) and identified conditionally dependent genes and gene groups at different resolutions. For a coarse resolution ($K=100$), VI-VS detected 26 groups of genes, seven of which contained genes known to be involved in the regulation of IL2RA [43], which we visualized in Fig. 6E. In addition to IL2RA and IL2RB, detections at the gene level included CCR5, which encodes a chemokine receptor that influences IL2 production in T cells [44]. Testing at several resolutions simultaneously identified causal genes that were not detected at the gene level, presumably due to sample size limitations or strong correlations. For instance, STAT3 and STAT5B, two transcription factors involved in the regulation of IL2RA, were not detected by VI-VS at the gene level but detected at a coarser resolution. STAT3 promotes T cell survival and is known to inhibit T cell proliferation and IL2 production [45]. The activation of STAT5B by IL2 cytokines is a critical signaling pathway associated with regulatory T cell differentiation and function [46]. We generalized this analysis to other proteins and compared the number of detected genes contained in known pathways for VI-VS and OLS more quantitatively (Fig. 6F). VI-VS detections at coarser resolutions detected more overlapping genes contained in pathways, while tests at finer resolutions provided more precise gene-level associations and overall more overlapping genes than OLS.

An important observation is that contrary to marginal approaches, VI-VS automatically controls for cell-type variation. A gene and a protein may be marginally dependent if they are expressed by the same cell types, even if they do not correlate within these types. Additional file 1: Figure S6 compares, for every gene, significance scores for its association with the surface protein CD4, using a marginal test and VI-VS with a significance score of DE between CD4+ T cells and the rest of the cells. The marginal approach has a very strong correlation with the cell-type variation, while VI-VS does not. Consequently, a marginal test for gene-protein association may reflect cell-type variation rather than molecular interactions. Conversely, since gene expression data from all genes except one are sufficient to identify cell types, VI-VS conditions the variation between cell types and finds associations that are not explained by cell-type variation.

### VI-VS identifies spatially dependent gene expression programs in lymphocytes using ST

We showcased how VI-VS can be applied for spatial transcriptomic (ST) analyses. In particular, we studied an ST dataset consisting of one lung biopsy from a non-small cell lung cancer (NSCLC) patient, containing 960 genes and 200,000 cells, sequenced using the CosMx platform [47] and segmented with Baysor [48] In this case, our objective was to link gene expression to spatial contexts that reflect cell localization in the tissue or its proximity to other cells.

#### Characterizing spatial differential expression patterns for T cells

**Fig. 7:**
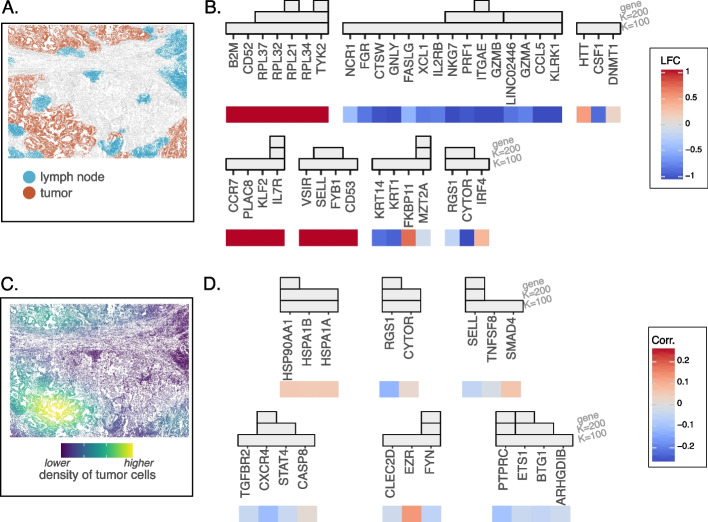
ST T cells experiment. **A** Tissue segmentation into lymph nodes and tumor regions. **B** Identified spatial DE genes by VI-VS in T cells, along with spatial LFCs (positive values denote gene upregulation in lymph nodes compared to tumors) against the significance scores of VI-VS. **C** Local density of tumor cells in the tissue. Density is estimated using kernel density estimation (bandwidth of 500 µm). **D** Identified T cell genes conditionally associated with local density of tumor cells, along with marginal Spearman scores between gene expression and local tumor density

We first aimed to identify differences in gene signature between T cells located in the tumor and lymphoid aggregates (Fig. 7A). We trained the considered models on these cells using a binary response variable indicating the cell location ($y=1$ for tumor cells, 0 for lymphoid aggregate cells); the importance score used by VI-VS was modified to the log-likelihood of a neural network binary classification model. We first compared the number of genes predicted by VI-VS to simple parametric and nonparametric differential expression tests (Additional file 1: Table S2). The latter approaches detected almost all genes in the dataset. As the number of cells increases, negligible differences in gene expression are likely to be detected as significant, even after multiplicity correction. This is a known problem for point null hypothesis tests applied to single cells [49, 50], which would require further filtering of the results to obtain a reasonable number of discoveries that can be interpreted. In contrast, VI-VS detected a much smaller number of genes at the gene level. Indeed, only five genes were detected by VI-VS at the gene level, including ITGAE and IL7R (Fig. 7B). ITGAE, encoding CD103, is a canonical marker of tissue-resident memory CD8+ T cells (Trm). Its expression characterizes T cell infiltration in the tumor microenvironment (TME) [51]. This result highlights that tissue-resident memory T cells preferentially infiltrate the tumor. IL7R is a general marker for memory CD4 T cells. The multiresolution approach detected a larger set of genes known to capture known tumor-specific T cell signatures. ITGAE is associated with a group of genes that have a cytotoxic function in CD8 cytotoxic T cells (CTSW, GNLY, NKG7, PRF1, GZMB, KLRK1) up-regulated in the tumor, identifying resident CD8 T cells in the tumor to have a highly cytotoxic and activated phenotype. IL7R on the contrary shows up in a module with CCR7 and KLF2, genes that mark central-memory CD4 T cells. This module therefore identifies central-memory CD4 T cells to be enriched outside of the tumor. These genes were mainly located in spatial clusters of lymphocytes which we identified as lymphoid aggregates. In general, the genes and modules detected reflect a diverse set of biological processes varying across lymphoid aggregates and tumor regions.

#### Identifying T cell genes associated with tumor proximity

Last, we characterized the associations between gene expression and tumor proximity by defining *y* as the local density of tumor cells surrounding T cells. The first step was to define the range of tumor proximity we considered relevant. To do so, we constructed several responses *y*, corresponding to the predicted tumor density for each T cell predicted by a Gaussian kernel density estimator of different bandwidths, taking values in $h \in [10\mu m, 50\mu m, 100\mu m, 200\mu m, 500\mu m, 1000\mu m]$. Of these, only $h=500 \mu m$ and $h = 1000\mu m$ detected associations at the gene resolution, suggesting that gene expression interactions with immediate and close tumor cells are more difficult to detect and would require more data to reach significance. We focused on the discoveries made by VI-VS for $h=500 \mu m$ (Fig. 7C), and visualized the detections, corresponding to six gene groups (Fig. 7D). Our approach provided a significant number of genes related to T cell function in the tumor microenvironment, including HSP90AA1, ETS1, CXCR4, RGS1, and FYN, all detected at the gene level. HSP90AA1, for instance, encodes a heat shock protein, whose overexpression correlates with tumor progression and a poor prognosis in NSCLC [52, 53], and has been shown to correlate with an exhausted phenotype of CD8 T cells in tumors [54]. CXCR4 encodes a chemokine receptor whose expression is associated with the formation of lymphoid follicles that we detected outside of the tumor [55]. TGFBR2 is associated with this gene at the module level and has been shown to induce the residency of T cells in lymphoid tissue [56]. A correlation of RGS1 with T cell exhaustion has been observed in various cancers, including NSCLC [57]. We emphasize here that the location of T cells outside of the tumor is related to specific chemokine signals, specific cell states, and markers of exhaustion, whereas the location inside the tumor is related to an increase in heat shock protein signatures associated with T cell exhaustion and cellular stress. These results suggest that VI-VS is flexible to be applied to continuous descriptions of spatial localization and then helps to dissect function without prior knowledge of important tissue niches.

## Discussion

Detecting conditional dependencies requires more data than identifying marginal dependencies [58]. This requirement may cause conditional approaches to miss potentially relevant associations due to limited statistical power. To address this risk, we proposed a multiresolution testing procedure. This procedure not only identifies individual genes with conditionally dependent features but also recognizes feature groups that may contain them, providing a comprehensive characterization of the statistical dependencies between features and responses. We highlight throughout the manuscript that these modules can help in identifying the functional role of an identified molecule and thereby help in interpreting the results.

In the general case, VI-VS discoveries have no guarantees to be functional. Feedback loops prevalent in molecular interactions, cell communication, or unobserved molecular species are just a few examples of phenomena that lead to spurious discoveries. However, certain multiomic setups already offer promising avenues for identifying causal relationships with VI-VS. Identifying reproducible and robust discoveries across biogically diverse environments could help mitigate the effect of unobserved confounders [59, 60].

Making no assumption on the distribution of the response, our approach can readily be applied to other multiomic setups. A first application could be the identification of gene associations with metabolites [61]. VI-VS could also be applied more broadly to spatial transcriptomics to create complex characterizations of cell phenotypes and their environments. It could, for instance, pinpoint genes involved in receptor-ligand interactions [62] or in determining cellular morphologies [63]. VI-VS is also relevant for the identification of potential transcription factor binding sites, using paired gene expression and chromatin accessibility data. A major advantage of VI-VS in this scenario is its ability to identify broader regulatory regions associated with gene expression, even when there is not enough data to pinpoint individual peaks.

The primary assumption underpinning VI-VS is the availability of a valid generative model of the feature data. The generative model should be capable of generating synthetic data that is statistically indistinguishable from the observed data. The generative model considered in this manuscript has undergone rigorous stress-testing for single-cell RNA data generation and imputation [64–66]. To show that VI-VS is not tied to this choice, however, we also showed that VI-VS also produced calibrated *p*-values using an alternative generative model for single-cell RNA-seq data (Additional file 1: Table S1). Other types of features may require the use of a generative model that better approximates the data generating process. For instance, chromatin accessibility data may require the use of a generative model that property account for ATAC-seq sparsity [67, 68] to ensure that VI-VS *p*-values remain valid. As illustrated in this work, more expressive importance scores, on the other hand, do not affect calibration but can improve power.

Technical data variations, such as differences in sample preparation and sequencing technologies, pose significant challenges for large-scale multiomic analysis [69, 70]. VI-VS effectively addresses this issue by conditioning on these nuisance factors. In single-cell studies, nonparametric tests, particularly the Conditional Randomization Test (CRT), have demonstrated the ability to produce calibrated significance scores in the presence of technical factors [71]. The generative models used by VI-VS are capable of capturing multiple nonlinear technical effects [72], enabling robust discoveries even in complex settings [73]. Therefore, we propose VI-VS as a general framework to produce calibrated significance scores of conditional associations in complex settings via integration of large datasets across multiple batches.

## Conclusions

VI-VS is a comprehensive framework for identifying potential functional relationships among molecular species in single-cell multiomics. It employs a nonparametric test for conditional independence, a concept that provides a more stringent notion of association than marginal tests. Unlike parametric tests, which require to posit a predefined relationship between features and the response, VI-VS does not require this relationship to be known. This makes VI-VS a versatile tool that remains valid even when the relationship between features and the response is unknown, promising to uncover novel insights into molecular and cellular interactions arising from multiomic measurements.

VI-VS can be employed as a meta-algorithm to make the discoveries of existing methods more interpretable by constructing importance scores from their predictions. In this work, we calibrated GENIE3 discoveries via VI-VS  but other models could be used instead. VI-VS is not as a replacement to such methods but rather as a wrapper algorithm that enables a principled and interpretable way to identify conditional dependencies with FDR control.

## Methods

As an input, VI-VS receives a matrix of features $X \in \mathbb {R}^{N \times G}$ and a vector, representing a response variable $y \in \mathbb {R}^{N}$ where *G* is the number of features and *N* is the number of cells. We also assume that observed nuisance factors $S \in \mathbb {R}^{N \times T}$, e.g., batch assignments, sequencing depths, or cell cycle events, affect these experiments and need to be accounted for. Our goal is to detect features in *X* that are associated with the response variable *y* while controlling for the nuisance factors.

In the following, we assume that *X* consists of observed molecular expressions of *G* genes in *N* cells, although the method is general and applies to other modalities. The choice of *y* varies depending on the assay considered and the problem of interest. Specifically, *y* can characterize molecular quantities, such as protein counts in CITE-seq experiments or chromatin accessibility in ATAC-seq data. It can also represent other, more abstract cell-level properties, e.g., characterizing the tissue environment of a cell in spatial transcriptomic assays. *y* may also correspond to a singled-out gene of interest, for which we wish to identify the interacting genes.

When referring to observations from an individual cell, we will employ lowercase letters, reserving uppercase letters for the entire array of observations. In addition, $x_g \in \mathbb {N}$ and $x_{-g} \in \mathbb {N}^{G -1}$ will respectively denote gene expressions for gene *g* and the vector of remaining genes. When needed, superscripts will index cells, such that $x^n$ denotes the gene expression vector $[x^n_1, \ldots , x^n_G]^T$ of cell *n*. When *A* is a set of features, $x_A$ will denote the vector of features contained in *A*. We make the assumption that the samples $(x^n, y^n, s^n)$ are independent and identically distributed (i.i.d.).

### Conditional randomization tests for single-cell genomics

To detect genes in *X* that are associated with the response variable *y*, VI-VS employs a *conditional* independence test, which estimates, for each gene, the plausibility of the null:

1 $$\begin{aligned} \mathcal {H}_{0, g} : x_g {\perp\mkern-10mu\perp} y \mid x_{-g}, s. \end{aligned}$$

We rely on the conditional randomization test (CRT) approach [30] to test these hypotheses. The premise of CRT is that while it is difficult to directly assess how the distribution of the response variable *y* depends on *X*, it is easier to describe how the features of *X* depend on each other. VI-VS requires two ingredients: a *generative model* for *X* to capture the dependencies between features and an *importance score* to evaluate their association with *y*.

#### Importance score

The importance score is a function $T: X, Y, S \rightarrow \mathbb {R}$, which summarizes the observed data. To make decisions, the CRT compares this summary *T*(*X*, *Y*, *S*), with $T(\tilde{X}, Y, S)$, where $\tilde{X}$ denotes partially synthetic data in which one or few of the features are replaced with values that are generated with the generative model. Here, we propose to *learn* the importance scores from the data. In particular, we consider importance scores corresponding to the prediction error of a regression model of *Y* on *X* and *s*,

2 $$\begin{aligned} T_\phi (X, Y, S) = \frac{1}{N} \sum \limits _{n=1}^N -\log p_\phi (y^n \mid x^n, s^n), \end{aligned}$$

where $(y^n, x^n, s^n)$ respectively denote responses, gene expression, and nuisance factors for cell *n*. Here, $p_\phi (y^n \mid x^n, s^n)$ is a likelihood for *y* based on a model $p_\phi$ trained on held-out data. For instance, $p_\phi$ may be based on a linear regression or more complex models such as random forest or a multi-layer perceptron (MLP). Importantly, this predictive model does not need to perfectly capture the conditional distribution of *y* given *x*, *s*. The CRT will indeed control the false positive rate irrespective of the choice of the predictor model and its assumptions on the nature of the interaction between *x* and *y* or on the distribution of *y* [30]. However, the more adequate the model, the more powerful we can expect the test to be.

#### Generative model

The other required component is a generative model $p_\theta$ that (i) can be used to sample *synthetic* expression profiles for a given gene and (ii) does not depend on the response variable *y*. Due to their scalability, ability to capture nonlinear effects, and flexible likelihood assumptions, latent variable models are a useful choice to model gene expression in this context. In these models, an unobserved low-dimensional variable *z* is assumed to capture the state of each cell and provide a concise summary of the biological variation among cells. We assume that the model factorizes, for each individual cell and under i.i.d. assumptions, as

3 $$\begin{aligned} p(x, z \mid s) = p(z) \left( \prod \limits _{g = 1}^Gp(x_g \mid z, s)\right) , \end{aligned}$$

where *p*(*z*) is the latent variable prior, and $p(x_g \mid z, s)$ is the likelihood for gene *g*. We rely on variational autoencoders (VAEs) to define the latent variable model. In this model, the prior is usually the standard normal, and the posterior distribution is approximated using a variational approach, with the approximation parameterized by neural networks [32, 74, 75]. Assuming access to such a model, testing $\mathcal {H}_{0, g}$ requires replacing the measurements for the feature *g*, with synthetic measurements that are conditionally independent of *y*. To this end, we use the generative model to obtain K vectors $\tilde{X}_g^{(k)}, k\le K$, containing synthetic counts for gene *g* for all the cells in a manner independent of *y*. Here, superscripts in parentheses denote Monte Carlo samples. We then construct the overall gene expression for which gene *g* was randomized, as

4 $$\begin{aligned} \tilde{\textbf{X}}^{(k)} := \left[X_1 \dots X_{g-1}, \tilde{\textbf{X}}_{\textbf{g}}^{\mathbf {(k)}}, X_{g+1}, \dots X_G\right]^T, ~~ 1 \le k \le K. \end{aligned}$$

With these two components, a *p*-value for $\mathcal {H}_{0, g}$ with the CRT corresponds to the proportion of random trials in which the importance score, when gene *g* is replaced with synthetic data, is not worse than the score obtained with the original data. It writes as

5 $$\begin{aligned} p_g = \frac{1}{K+1} \left( 1 + \sum \limits _{k=1}^K \mathbb {I} \left( T(\tilde{\textbf{X}}^{(k)}, Y, S) \le T(\textbf{X}, Y, S)\right) \right) . \end{aligned}$$

### Valid inference for CRTs with latent variable models

Given a latent variable model, an intuitive way to generate synthetic samples $\tilde{X}_g$ is by independent draws from the Gibbs distribution:

$$\begin{aligned} \tilde{x}_g^n \sim p_\theta (x_g^n \mid x^n_{-g}, s) = \int p(x_g \mid z) p(z \mid x_{-g}^n, s)dz. \end{aligned}$$

This choice, however, requires sampling from $p(z \mid x_{-g}, s)$. In the context of VAEs, this requires training a separate model for every feature *g*, which is in most cases computationally prohibitive. Instead, VI-VS provides a fast and valid sampling alternative that still provides valid *p*-values. This is done by drawing *fixed* posterior sample of *z*. Here, for each cell *n*, we first sample one particle from $\bar{z} \sim q(z \mid x^n)$ where *q* is the encoder network of the VAE. We then rely on the decoder network of the VAE to obtain synthetic samples:

6 $$\begin{aligned} \tilde{x}^{(k)}_g \overset{\text {iid}}{\sim } p_\theta (x_g \mid z=\bar{z}, s),\quad k \le K. \end{aligned}$$

Note that in both cases the generative model does not have access to the value of *y* during sampling, The samples $\tilde{\textbf{X}}^{(k)}$ therefore reflect a hypothetical reality in which $x_g$ and *y* are conditionally independent. In Proposition 1 we demonstrate that both sampling schemes provide valid *p*-values for the CRT (proof in Additional file 1).

#### Proposition 1

(Valid sampling distributions for CRTs with latent variable models). Assume a latent variable model $p_\theta (x, z \mid s)$, factorizing as (3). Let $\tilde{X}_g = [\tilde{x}_g^1, \dots \tilde{x}_g^N]^T$ be a vector of synthetic gene expression profiles generated using the latent variable model for gene *g* obtained using either of the two sampling schemes described above. Then, the *p*-values $p_g$ in Equation (5) have a distribution that stochastically dominates the uniform distribution when the null hypothesis $\mathcal {H}_{0, g}$ holds. That is, $p_g$ is a valid *p*-value.

The entire procedure can therefore be summarized as follows:

**Figure:**
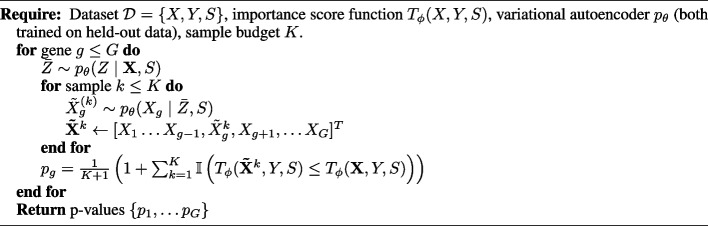
**Algorithm 1** Conditional randomization tests with VI-VS

VI-VS further corrects the obtained *p*-values $p_g$ using the Benjamini-Hochberg procedure [76] to control the false discovery rate (FDR), described in Additional file 1: Supplement C1.

#### Cell-specific scores

Equation (5) quantifies the significance of the association between gene *g* and protein *p*; it does not, however, inform on which cell subpopulations may be most responsible for this association. For this purpose, we introduce the cell-specific score,

7 $$\begin{aligned} s_g(x, y, s) := \frac{1}{K} \left[ \sum \limits _{k=1}^KT(\tilde{x}^{(k)}, y, s)\right] - T(x, y, s), \end{aligned}$$

where $\tilde{x}^{(k)}$ denotes a randomized sample for the CRT. In other words, positive scores will highlight that randomizing the gene *g* in cell *x* increases the predictive loss, which may mean that this gene plays a relevant role in the prediction *y* for the considered cell.

### Multi-resolution hypothesis testing

Conditional dependence is a more stringent statistical notion than marginal dependence. Consequently, applying conditional independence tests at the gene level may not yield many significant genes. This could be due to several factors, such as limited sample sizes or strong correlations between genes that make it challenging to reject the conditional null.

In scenarios with small sample sizes it might prove challenging to detect a true positive gene if it heavily correlates with other genes in its cluster. However, it is easier to detect that one or more genes in the cluster are conditionally associated with the response, even if we cannot definitively identify which genes are responsible.

Therefore, following an approach introduced for genome-wide association studies [77], we test for conditional independence at multiple resolutions, ranging from broad groups of genes to the individual gene-level resolution, to avoid overlooking genes of interest.

To further illustrate how VI-VS behaves as correlation between features increases, we devised a simple simulation study, where a synthetic response *Y* is conditionally dependent with one feature *g*, which itself correlates with another feature $g'$ (Additional file 1: Figure S7). Briefly, as the correlation between features increases, it is not possible to detect the conditionally dependent relationship between *Y* and *g* anymore. However, at a coarser resolution, the group of genes *g* and $g'$ is still detected as conditionally dependent with *Y*.

We will now explain how we (i) group genes together and (ii) test for conditional independence of a group of genes.

#### Determining relevant clusters of genes

Our goal is to group together features associated with the same biological functions. We assume that high correlations between features may indicate that they are associated with the same biological function. Consequently, we cluster genes based on their empirical correlation matrix. Any gene clustering algorithm can be used in principle, e.g., [78]. We propose using a fast hierarchical clustering approach that is scalable to large datasets. This approach performs agglomerative clustering based on the gene-by-gene empirical correlation matrix computed on the normalized gene expression of the generative model, e.g., scVI [32]. More details about this procedure can be found in Additional file 1: Supplement D.2. At a specified resolution *K*, the clustering provides a partition *M* of all genes into *K* groups of genes $A_1, \dots , A_K$.

#### Group conditional independence

Next, we aim to determine whether a cluster of genes is significant. To formalize this, let $A \in M$ denote a group of genes. We are interested in interactions of the form

8 $$\begin{aligned} \mathcal {H}_{0, A}^M : x_{A} {\perp\mkern-10mu\perp} y \mid x_{A^C}, \end{aligned}$$

where $x_A$, $x_{A^C}$ denote the gene expression vectors for the genes in sets *A* and its complement $A^C$, respectively. We test this null hypothesis using the same procedure as described above, with more details provided in Additional file 1: Supplement D.2 and illustrated in Additional file 1: Figure S8. Specifically, we can test Eq. 8 by sampling from the same distribution as in Algorithm 1.

### Faster inference using parallel computing

We implemented VI-VS in a fast and scalable way that is available as an open-source Python package. The scalability of this solution relies on two components. Our implementation first relies on parallel computing and just-in-time compilation components of Jax to speed up the inference, allowing us to efficiently compute the *p*-values for all genes. This practical choice offered a twofold speedup compared to a Pytorch backend (Additional file 1: Figure S9). The second key component is the fact that we have set up our algorithm to avoid fitting a model for each Monte Carlo sample, and instead, we only have to perform a forward pass of the pre-fit feature statistic. This computational ingredient improves the run time by orders of magnitude, thus improving scalability.

## Supplementary Information

Additional file 1. Theoretical background and proof of Proposition 1; Supplementary Information of VI-VS and on the experiments; additional experiments.

Additional file 2. Review history.

## Data Availability

VI-VS is available as a Python package on PyPI or on GitHub at https://github.com/YosefLab/VIVS under the permissive BSD-3-Clause License [79]. The source code to reproduce the results in this paper is available at https://github.com/PierreBoyeau/VIVS-reproducibility and on Zenodo at DOI: 10.5281/zenodo.13323809 [80].
