# Supplementary material for: VI-VS: calibrated identification of feature dependencies in single-cell multiomics
Source: Genome Biol. 2024 Nov 15;25:294. doi: 10.1186/s13059-024-03419-z (PMC11566124; doi:10.1186/s13059-024-03419-z)
Supplement: Supplementary file 1 — Additional file 1. Theoretical background and proof of Proposition 1; Supplementary Information of VI-VS and on the experiments; additional experiments. [file 13059_2024_3419_MOESM1_ESM.pdf]

Supplementary Information

Contents

A Supplementary Information on the experiments **1**
A.1 Models . . . 1
A.2 Semi-synthetic data . . . 2
A.3 Perturb-seq experiment . . . 2
A.4 CITE-seq experiment . . . 2
A.5 Nanostring experiment . . . 2

B Additional results **3**
B.1 Semisynthetic Poisson experiment . . . 3
B.2 Influence of key parameters on the semi-synthetic experiment . . . 3
B.3 Alternative generative model in the semi-synthetic experiment . . . 3
B.4 Effect of the development set size on the semi-synthetic experiment . . . 4
B.5 Perturb-seq dataset . . . 4
B.6 Additional CITE-seq experiments . . . 4
B.7 Comparison of the number of significant hits for the spatial DE analysis . . . 6

C Theoretical background and guarantees **6**
C.1 False Discovery Rate control . . . 6
C.2 Proof of Proposition 1 . . . 6

D Supplementary Information on `VI-VS` **7**
D.1 Feature-level and group-level conditional independence as a function of feature correlations . 7
D.2 Multiresolution analysis . . . 7
D.3 Implementation of `VI-VS` . . . 8
D.4 Generalization to multidimensional Y . . . 8
D.5 Using `VI-VS` as a way to calibrate an existing feature selection method . . . 9

A Supplementary Information on the experiments

A.1 Models

`VI-VS` We trained both scVI and the model associated with the importance scores until convergence using early stopping criteria on the held-out validation data.

OLS and marginal tests We computed OLS and marginal tests from log-CPM normalized counts. Tests relative to OLS and to the marginal baseline were fitted using the *statsmodels* python package.

Multiplicity control P-values obtained with `VI-VS` OLS, and the marginal test were adjusted using the Benjamini-Hochberg procedure [1].

A.2 Semi-synthetic data

We consider a PBMC scRNA-seq dataset consisting of $N = 6,855$ cells and $G = 500$ genes. We construct 5-dimensional synthetic surface protein measurements using the following scheme. Let $p \leq 5$ be some protein. We first construct the subset of conditionally dependent genes S using Binomial sampling, with sparsity rate $s = 0.3$. Mean measurements for protein p are obtained as

$$\mu_p = A_p \tilde{x}_S + \epsilon_p, \tag{1}$$

$A_p \in \mathbb{R}^{N \times s}$ corresponds to the ground-truth regressors, constructed as $A_p = \beta B$, where B denotes the $N \times s$ matrix of ones and β is the signal strength, parameterized as

$$\beta = \beta_0 \sqrt{\frac{2 \log G}{N}}. \tag{2}$$

A.3 Perturb-seq experiment

Data preprocessing We restricted the analysis to ten targeted genes with the highest number of single-guide RNA (sgRNA) perturbations. We identified true positive associations using a t-test on log-normalized counts comparing gene expression between cells without guide RNA, and cells with guides targeting the relevant gene. In particular, true positives interactions were defined as interactions for which multiplicity-adjusted p-values were below 0.1. We also restricted the analysis to 1000 pseudo-genes using the following procedure. We first clustered the genes using k-means based on their empirical correlation matrix, computed on log-CPM counts. For each obtained cluster, we summed the expression of the genes in the cluster, and used the resulting quantity as a pseudo-gene. A pseudo-gene was considered as a true positive if it contained at least one gene that was significantly differentially expressed between cells with and without guide RNA.

A.4 CITE-seq experiment

Data preprocessing The original data [2] contained gene expression and surface protein counts for eight donors at several time points, corresponding to both pre-vaccination and post-vaccination. We focused our analysis on the pre-vaccination time points, resulting in a dataset containing a total of 50,000 cells and 2,000 genes. These genes were selected based on a procedure hoping to retain as much information about cells' biological states as possible. To do so, we selected genes based on their joint variability with other genes instead of their marginal variability. To do so, we first clustered the genes using k-means based on their empirical correlation matrix, computed on log-CPM counts. In each cluster, we selected the gene with the highest highly variable-gene score, as predicted by Seurat [3]. The surface protein measurements y were centered and scaled to have zero mean and unit variance.

A.5 Nanostring experiment

Data segmentation and annotation Baysor [4] was used to segment the tissue into individual cells. We downloaded the lung FFPE Nanostring CosMX data. We used the position file of Lung 5 replicate 2 and split this into separate files for each FOV. Inside each FOV, we used the provided segmentation from Nanostring as a prior for Baysor segmentation and chose 0.1 as the prior segmentation weight. In short, we multiplied the z-position of each molecule by 10 to adjust for anisotropic distances. These results were concatenated across all FOV and scVI [5] was used to integrate these field of views. Leiden clustering was performed after neighbor calculation in latent space and cell-types were annotated based on known marker genes. We focused our analysis on T cells.

Identification of T cell markers in spatial analysis A number of genes not expressed in T cells might still be detected in the T cell analysis, due to contamination events, e.g., due to imperfect cellular segmentation. To circumvent this issue, we restrict the analysis of the spatial datasets to genes that are

higher expressed in T cells compared to other cell-types. We identified these genes via one-vs-all differential expression analysis based on log-CPM counts, using t-tests to compare mean expression levels, and BH to correct for multiple testing. We then constructed a list of 245 genes, containing all genes with ajusted p-values < 0.05, and with a log-fold change > 0.1 (upregulated in T cells).

B Additional results

B.1 Semisynthetic Poisson experiment

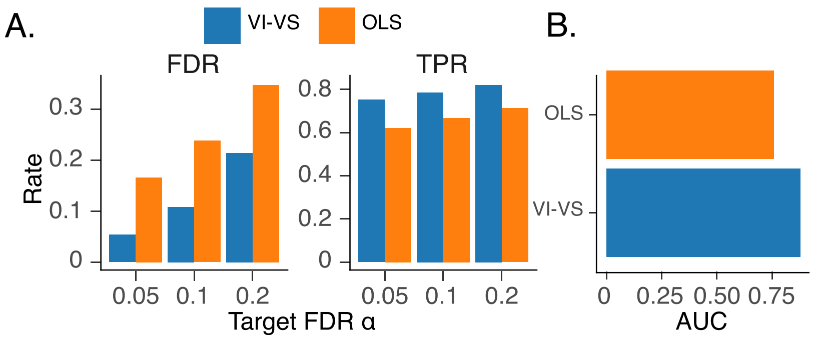

Figure S1: Semisynthetic experiment from a Poisson model. **A.** *Left*: FDR levels reached by `VI-VS` and OLS. *Right*: Power levels reached by `VI-VS` and OLS. **B.** Comparison of the area under the precision-recall curves of the different algorithms.

We also considered a scenario where protein expressions suffer from Poisson instead of Gaussian noise, and where the relationship the protein expressions means are a linear combination of *squared* gene expressions. In such a scenario, an approach based on OLS does not produce valid p-values because of the model assumptions' violations, while our approach still returns valid p-values (Figure S1). The power of our approach remains high, and has an higher area under the precision-recall curve to OLS.

B.2 Influence of key parameters on the semi-synthetic experiment

Contrary to OLS, the CRT-based approach provides consistently calibrated predictions (Figure S2). We however note that our approach displays intriguingly high FDR values for different sparsity levels. These scenarios correspond to regimes where the test detects a handful of discoveries.

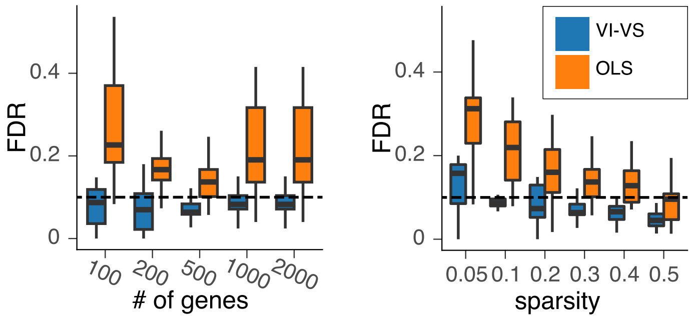

Figure S2: Influence of several parameters of importance in the semi-synthetic experiment. *Left*: Influence of the number of observed genes in the experiment. *Right*: Influence of the sparsity of the ground-truth parameter β.

B.3 Alternative generative model in the semi-synthetic experiment

We also considered an alternative generative model to scVI to produce synthetic gene expression counts. We considered a linear generative model, ldVAE, using a negative binomial likelihood, corresponding to [6].

The model was reimplemented in `jax`. Table S1 displays the obtained FDR and power for both scVI and linear generative model. These results show that the linear generative model controls the FDR, at a similar power level to scVI. This observation suggests that a simpler generative model suffices to produce valid p-values in this scenario. This experiment also shows that `VI-VS` is robust to the choice of generative model.

Table S1: Comparison of the FDR and power for the linear generative model and scVI on the semi-synthetic experiment for five random seeds, for a target FDR of $\alpha = 0.1$.

	**scVI**	**ldVAE**
FDR	0.06 ± 0.03	0.06 ± 0.03
Power	0.93 ± 0.03	0.94 ± 0.03

B.4 Effect of the development set size on the semi-synthetic experiment

We studied the effect of the development set size on the semi-synthetic experiment described in Supplement A.2. We observed that for all development set sizes above 0.5, `VI-VS` provided calibrated pvalues with high power, showing that `VI-VS` is robust to the train-test validation side. These development set sizes corresponded to splits for which the validation negative evidence lower-bound (ELBO), which is a proxy for the model's fit, was low. These splits occur after the ELBO curve's inflection point, where the model's fit stabilizes and shows minimal improvement with additional data.

Consequently, we recommend, when required, the following heuristic for the choice of the development set size. When possible, a good heuristic is to fit `VI-VS`'s generative models for a range of development set sizes, and to pick a development set size after the ELBO curve's inflection point.

A major benefit of `VI-VS` is that FDR calibration be assessed once the generative model has been fitted. For this purpose, a synthetic response variable can be generated using similar strategies as employed in Supplement A.2, and calibration can be assessed using the synthetic response variable before considering the real response variable.

Absence of FDR control on such an experiment is a sign that the generative model does not properly capture features, and that further hyperparameter tuning of the generative model (including the development size) might be necessary to ensure a proper fit of the generative model to the data.

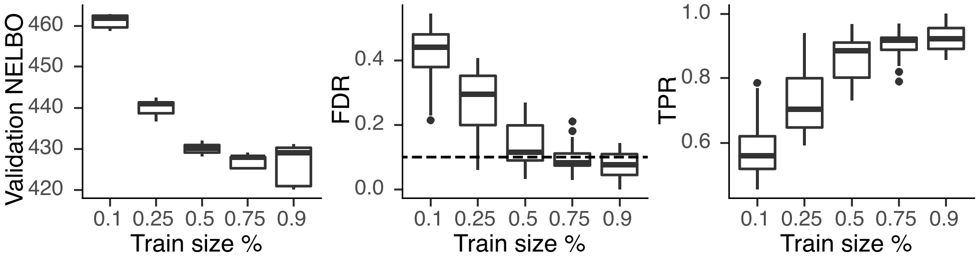

Figure S3: Effect of the development set size of `VI-VS` on the semi-synthetic experiment based on five random seeds for each size. *Left*: Validation negative evidence lower-bound (lower is better). *Middle* and *Right*: FDR and TPR for `VI-VS` decisions for a target FDR at level $\alpha = 0.1$.

B.5 Perturb-seq dataset

Figure S4 shows additional metrics for the decision obtained by the different algorithms on the perturb-seq dataset.

B.6 Additional CITE-seq experiments

Figure S5 shows that the proteins for which `VI-VS` detects conditional associations have a higher percentage of cells expressing the protein.

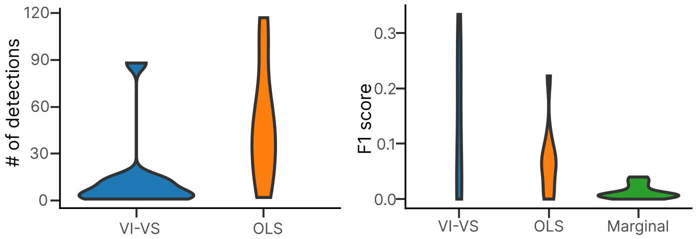

Figure S4: Additional metrics for the perturb-seq dataset, for the same decisions as in the main text. *Left*: Number of detections produced by the different algorithms. Marginal, similar to the rest of the experiments, detected a much higher number of hits (not shown). *Right*: F1-scores for the different algorithms.

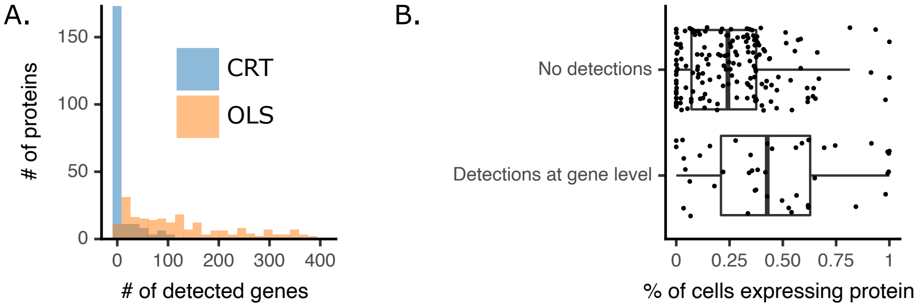

Figure S5: **A**. Comparison of the number of detections made by `VI-VS` and OLS across all proteins. **B**. Comparison of the percentage of cells expected to express the proteins for proteins for which (i). `VI-VS` predicts associated genes, and (ii). `VI-VS` does not predict associated genes. We predicted the percentage of cells expressing the proteins using TOTALVI, defining that a cell expressed a protein when the posterior probability observed protein counts comes from the foreground mode was above 0.95. Differences between the two groups are statistically significant (p-value $< 10^{-4}$ under a Kolmogorov–Smirnov test). In both these experiments, a gene-protein association is considered significant when the adjusted p-value is below 0.1.

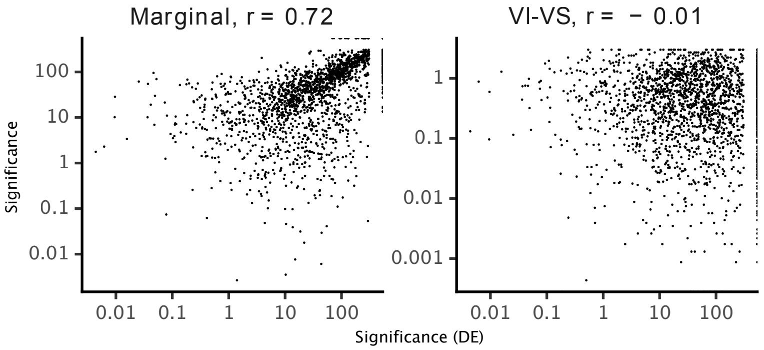

Figure S6: Comparison of significance scores for a Wilcoxon test between CD4+ and CD4- cells (*x-axis*) gene-protein significance scores (*y-axis*) for both a marginal test (*left*) and `VI-VS` (*right*). Significance scores were obtained as negative log p-values for the various tests. Correlation scores were computed using Spearman's rank correlation.

B.7 Comparison of the number of significant hits for the spatial DE analysis

Table S2 compares the number of significant hits for all considered methods for the spatial DE analysis.

	At cluster level	At gene level
VIVS	40	5
t-test	$\emptyset$	216
Wilcoxon	$\emptyset$	218

Table S2: Comparison of the number of significant hits for the spatial DE analysis for VI-VS as well as common DE approaches.

C Theoretical background and guarantees

C.1 False Discovery Rate control

FDR control aims at ensuring that the rate of false positive detections made in multiple hypothesis testing scenarios is controlled in expectation. Formally, the FDR characterizes the expected proportion of erroneous discoveries:

$$FDR := \mathbb{E}\left[\frac{|S \cap N|}{\max(1, |S|)}\right], \tag{3}$$

where S denotes the set of detected associations, and N the set of non-existing associations in the data.

C.2 Proof of Proposition 1

Proposition 1 is a consequence of the following theorem,

Theorem 1 (CRT gives valid inference, from Lemma 4.1 in [7])**.** *Let $\tilde{x}_g$ denote some synthetic sample for feature g from some proposal distribution (a distribution used to generate synthetic samples). Suppose under $\mathcal{H}_{0,g}$, $\tilde{x}_g$ satisfy the following property:*

$$(\tilde{x}_g, x_1, \dots x_g, \dots x_G, y) \overset{d}{=} (x_g, x_1, \dots \tilde{x}_g, \dots x_G, y) \mid s, \tag{4}$$

for $k = 1, \dots, K$. Above, the notation $\overset{d}{=}$ denotes equality in distribution conditioned to s. If $\tilde{x}_g^{(1)}, \dots \tilde{x}_g^{(K)}$ are K i.i.d. draws from this proposal distribution, then,

$$p_g := \frac{1}{K+1}\left(1 + \sum_{k=1}^{K} \mathbb{I}\left(T(\tilde{\mathbf{x}}^{(k)}, y) \leq T(\mathbf{x}, y)\right)\right),$$

where $\tilde{x}^{(k)} = [x_1, \dots \tilde{x}_g^k, \dots x_G]$, $k \leq K$, is a valid p-value for $\mathcal{H}_{0,g}$ (i.e., for the conditional independence test).

Property (4) ensures that the distribution of the data remains the same even when the true and synthetic samples are swapped. Equation 4 is a pairwise exchangeability property that ordered sets $(\tilde{x}_g, x_1, \dots x_g, x_G, y)$ and its swapped counterpart $(x_g, x_1, \dots \tilde{x}_g, x_G, y)$ have the same distribution. In particular, this property is a relaxation of the exchangeability propert required to construct knockoffs. We refer to [7] for a more detailed discussion and interpretation of this property.

Proof. Assume that the distribution of synthetic samples $\tilde{x}_g$ satisfy Property (4) under the null. Then, conditional on s, under the null we have

$$T([x_1, \dots x_g, \dots x_G], y) \overset{d}{=} T([x_1, \dots \tilde{x}_g, \dots x_G], y).$$

Since $\tilde{x}_g^{(1)}, \dots \tilde{x}_g^{(K)}$ are i.i.d draws from the conditional distribution and x_g is an independent sample from the same conditional distribution, we conclude

$$\frac{1}{K+1}\left(1 + \sum_{k=1}^{K} \mathbb{I}\left(T(\tilde{\mathbf{x}}^{(k)}, y) \leq T(\mathbf{x}, y)\right)\right) \sim \text{Uniform}\left(\frac{1}{K+1}, \frac{2}{K+1}, \dots 1\right). \tag{5}$$

□

We now use this result to prove Proposition 1, and in particular, that the two described sampling schemes verify the exchangeability property required to apply Theorem 1.

Proof. Proposition 1 states that two sampling schemes are valid for the CRT. The first sampling scheme corresponds to sampling from the distribution given by $\int p(x_g \mid z, s)p(z \mid x_{-g}, s)dz$. From our assumptions on the generative model, we have that

$$p(x_g \mid x_{-g}, s) = \int p(x_g \mid z, s)p(z \mid x_{-g}, s)dz.$$

Hence, (4) is satisfied. (See [7] for a more detailed proof.)

We now consider the second sampling scheme, which is the case presented in Algorithm 1. We let $\bar{z} \sim p(z \mid x, s)$ be a latent variable sampled from the posterior distribution. Let $x := [x_1, \dots x_g, \dots x_G]$ denote the observed gene expression profile. For clarity in the following equations, the subscript notation is used to specify which random variable is being considered. For example, $p_{x_g|z,s}$ represents the density of random variable x_g given z and s.

Under the null, we have the following joint distribution

$$\begin{aligned} p_{\tilde{x}_g,x,y,\bar{z}|s}(\tilde{x}_g, x, y, \bar{z} \mid s) &= p_{x_g|z,s}(\tilde{x}_g \mid \bar{z}, s)p_{x|s}(x \mid s)p_{z|x,s}(\bar{z} \mid x, s)p(y \mid x_{-g}, s) \\ &= p_{x_g|z,s}(\tilde{x}_g \mid \bar{z}, s)p_{x_g|z,s}(x_g \mid \bar{z}, s)p_{x_{-g}|z,s}(x_{-g} \mid \bar{z}, s)p_z(\bar{z})p(y \mid x_{-g}, s) \end{aligned} \tag{6}$$

These equalities follow from the assumed factorization of the generative model and from Bayes' rule. Notice the last expression is symmetric in the arguments x_g and $\tilde{x}_g$. We conclude that x_g and $\tilde{x}_g$ have the same distribution conditional on s and $\bar{Z} = \bar{z}$. We can thus apply Theorem 1 with conditioning on (s, $\bar{z}$).

□

Observed that exchangeability property does not hold unconditional to a fixed value of z. This can be seen in Equation 6, since $p(z \mid [x_1, \dots \tilde{x}_g, \dots x_G])$ and $p(z \mid [x_1, \dots x_g, \dots x_G])$ are not equal in general. This justifies why z must be fixed when sampling $\tilde{x}_g$, and cannot be sampled from the posterior distribution at every MC trial.

D Supplementary Information on `VI-VS`

D.1 Feature-level and group-level conditional independence as a function of feature correlations

We considered a simple simulation to illustrate how `VI-VS` behaves as the correlation between features increases. To do so, we considered the data from Supplement A.2. Starting from a random gene g, we constructed a synthetic gene expression g' that correlated with g,

$$X_{ng'} = X_{ng} + \epsilon_n,$$

where n denotes the cell index. Here $\epsilon_1, \dots, \epsilon_N$ are i.i.d. Poisson draws from a Poisson distribution with parameter λ. As λ decreases, the correlation between g and g' increases. The response Y was generated using the same procedure as in Supplement A.2, such that g was conditionally dependent with Y. Figure S7 displays the obtained pvalues for the conditional association between X_g and Y as a function of λ. We observed that in a regime where the correlation between X_g and X'_g is high, it was not possible to detect the conditional association between X_g and Y. At a coarser level, however, the conditional independence test was able to detect the conditional association. These observations highlight a potential limitation of feature-level conditional independence tests, that the multiresolution component of `VI-VS` is able to overcome.

D.2 Multiresolution analysis

In this section, we describe how `VI-VS` performs multiresolution analysis.

Feature clustering. The first step of this procedure consists in clustering features. While any clustering procedure can be used, we rely on a custom hierarchical clustering procedure when features are genes. After fitting scVI on all genes, we compute cell and gene specific denoised gene expresssion levels from the fitted model. We then compute the empirical correlation matrix between genes. We then used complete-linkage clustering to cluster genes at arbitrary resolution.

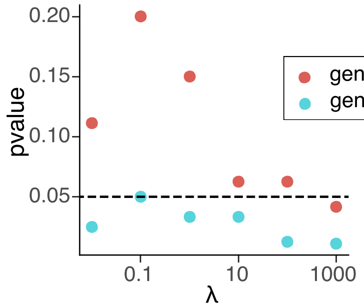

Figure S7: Obtained pvalues for the conditional association between X_g and Y as a function of λ. The correlation between X_g and X'_g increases as λ decreases.

Multiresolution testing. Testing for conditional independence at the cluster level follows the same procedure as in the main text. In particular, let A denotes a set of genes for which we want to test

$$\mathcal{H}_{0,A}^{M} : x_A \perp\!\!\!\perp y \mid x_{A^C}.$$

We first randomize the expression for all genes in A, obtaining expressions $\tilde{X}_A = \{\tilde{X}_g, g \in A\}$, where $\tilde{X}_g$ is obtained from Equation 6. We then construct the randomized expression profile for all genes,

$$X' = \begin{cases} X & \text{if } g \notin A \\ \tilde{X}_g & \text{if } g \in A \end{cases} \tag{7}$$

We then compute the randomized test statistic as $T(X', Y, S)$. We then repeat this procedure K times, obtaining K randomized test statistics $T^{(1)}, \ldots T^{(K)}$, which we compare with the actual test statistic to construct the p-value for the conditional null.

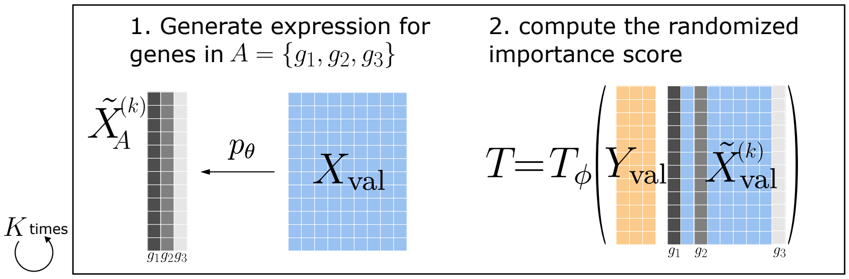

Figure S8: Illustration of the randomization procedure when testing at the gene group level. We rely on the generative model to produce synthetic gene expression profiles for all genes in the group A. Then, the randomized values are replaced in the original gene expression matrix to compute the randomized expression scores. This procedure is repeated K times to obtain the p-value for the conditional null.

Computational considerations. `VI-VS` relies on an efficient implementation that allows one to compute p-values across multiple resolutions in parallel. In particular, we use the same synthetic samples across all resolutions.

D.3 Implementation of `VI-VS`

We implemented `VI-VS` in python using Jax as backend. In particular, we relied on Algorithm S1 to compute the p-values, allowing to efficiently compute p-values using GPU acceleration. Our implementation relies on just-in-time compilation and parallelized computation to achieve high performance. This choice provides a two-fold improvement over an implementation relying on Pytorch, that benefits from a similar GPU acceleration but does not support just-in-time compilation nor parallelized computation.

D.4 Generalization to multidimensional Y

While the main text of this work assumes that Y is unidimensional, we here outline how to handle cases where the measurement is D-dimensional. To do so, we consider a multivariate regression task to fit the

Algorithm S1 Minibatched `VI-VS`

Require: Collection of B minibatches $\mathcal{D} = \{(X', Y', S')_{b \leq B}\}$, data statistic $T(X, Y, S)$, Sample budget K, trained generative model $p_\theta(X)$.
Compute $T_{\text{obs}} := T(\mathbf{X}, Y, S)$
for gene $g \leq G$ **do**
  $T^k \leftarrow 0, \ \forall k \leq K$
  for cell minibatch X', Y', S' **do**
    for sample $k \leq K$ **do**
      Use p_θ to sample $\tilde{X}_g'^k$
      $\tilde{\mathbf{X}}'^k \leftarrow [X_1' \dots X_{g-1}', \tilde{X}_g'^k, X_{g+1}', \dots X_G']^T$
      $T_k \leftarrow T_k + T(\tilde{\mathbf{X}}'^k, Y', S')$
    end for
  end for
  $p_g = \frac{1}{K+1}\left(1 + \sum_{k=1}^K \mathbb{I}\left(T(\tilde{\mathbf{X}}^k, Y, S) \leq T(\mathbf{X}, Y, S)\right)\right)$
end for
Return p-values $\{p_1, \dots p_G\}$

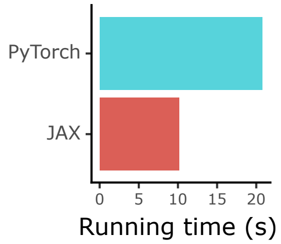

Figure S9: Running time comparisons. Execution time of `VI-VS` on a single GPU (RTX 3090) to compute p-values on a dataset containing 6,000 observations and 500 genes for 100 MC trials.

importance score. We train its parameters via optimization of the data likelihood, which writes for a single observation as $\mathcal{L}_\phi(y, x, s) := \sum_{d=1}^D \log p_\phi(y_d \mid x, s)$, assuming conditional independence of the features of y.

Two scenarios can appear when performing conditional independence testing with `VI-VS` in a multidimensional setup. First, it can be desirable to retrieve conditional dependencies specific to each feature of y,

$$\mathcal{H}_{0,g}^d : y_d \perp\!\!\!\perp x_g \mid s. \tag{8}$$

In this case, we use $T_\phi^d(X, Y, S) := \frac{1}{N}\sum_{n=1}^N -\log p_\phi(y_d^n \mid x^n, s^n)$ as importance scores to test $\mathcal{H}_{0,g}^d$.

It can also be the case that we would like to test

$$\mathcal{H}_{0,g} : y \perp\!\!\!\perp x_g \mid s, \tag{9}$$

where in this case y is multidimensional. In this scenario, we use the full likelihood of the predictive model p_ϕ as importance scores.

D.5 Using `VI-VS` as a way to calibrate an existing feature selection method

`VI-VS` can be used to calibrate some existing feature selection methods that would not provide significance scores. Methods which rely on a regression fit to the data to ultimately score interactions between features and the response can straightforwardly be calibrated with `VI-VS`. Such methods include, for instance, regularized linear regression models for which significance scores are not easily available [8, 9], or ensembling methods [10, 11], that are popular feature selection tools employed for GRN inference.

For such methods, we first fit the regression model f on the development split, and define the importance score for the CRT as the mean prediction error of the model:

$$T(X, Y) := \frac{1}{N} \sum_{n=1}^{N} (y^n - f(x^n))^2 \tag{10}$$

References

1. Benjamini, Y. & Hochberg, Y. Controlling the False Discovery Rate: A Practical and Powerful Approach to Multiple Testing. *The Journal of the Royal Statistical Society, Series B* **57,** 289–300 (1995).
2. Hao, Y. *et al.* Integrated analysis of multimodal single-cell data. *Cell* **184,** 3573–3587 (2021).
3. Stuart, T. *et al.* Comprehensive integration of single-cell data. *Cell* **177,** 1888–1902.e21 (June 2019).
4. Petukhov, V. *et al.* Cell segmentation in imaging-based spatial transcriptomics. *Nature Biotechnology* **40,** 345–354 (Mar. 2022).
5. Lopez, R., Regier, J., Cole, M. B., Jordan, M. I. & Yosef, N. Deep generative modeling for single-cell transcriptomics. *Nature Methods* **15,** 1053–1058 (2018).
6. Svensson, V., Gayoso, A., Yosef, N. & Pachter, L. Interpretable factor models of single-cell RNA-seq via variational autoencoders. *Bioinformatics* **36,** 3418–3421 (2020).
7. Candes, E., Fan, Y., Janson, L. & Lv, J. Panning for gold:'model-X'knockoffs for high dimensional controlled variable selection. *Journal of the Royal Statistical Society: Series B (Statistical Methodology)* **80,** 551–577 (2018).
8. Zou, H. & Hastie, T. Regularization and variable selection via the elastic net. *Journal of the Royal Statistical Society* **67,** 301–320 (Apr. 2005).
9. Tibshirani, R. Regression shrinkage and selection via the lasso. *Journal of the Royal Statistical Society* **58,** 267–288 (Jan. 1996).
10. Huynh-Thu, V. A., Irrthum, A., Wehenkel, L. & Geurts, P. Inferring regulatory networks from expression data using tree-based methods. *PLoS One* **5** (Sept. 2010).
11. Moerman, T. *et al.* GRNBoost2 and Arboreto: efficient and scalable inference of gene regulatory networks. *Bioinformatics* **35,** 2159–2161 (June 2019).
