# Supplementary material for: VI-VS: calibrated identification of feature dependencies in single-cell multiomics
Source: Genome Biol. 2024 Nov 15;25:294. doi: 10.1186/s13059-024-03419-z (PMC11566124; doi:10.1186/s13059-024-03419-z)
Supplement: Supplementary file 2 — Additional file 2. Review history. [file 13059_2024_3419_MOESM2_ESM.docx]

**1st round**

**Reviewer 1**

Boyeau et al. introduces a new framework, VI-VS, to explore conditionally dependent features using variational inference. VI-VS quantifies the credibility of pairwise interactions of the observed features compared to the synthetic ones, without making assumptions about the distribution of the response variables and is able to capture the non-linear relationship between variables using a neural network framework. Overall, this paper proposed a very interesting formulation and framework to identify feature dependencies in single-cell datasets.

I have the following comments of the manuscript.

1. The methods are based on the previous works of ModelX knockoffs. I am wondering how well using generative model scVI preserve the dependencies between features. How would other ways to construct synthetic gene expression matrix perform compared to scVI, such as scDesign3, scGAN and ZINB-WaVE?

2. How stable is the output of VI-VS for different splits of datasets?

3. Following the last point, how reproducible the results of VI-VS when applying to different batches of data? For example, if there are two technical replicates of spatial data from or different batches of scRNA-seq data, can VI-VS identify similar features?

4. How would VI-VS compare to other non-linear machine learning methods that are commonly used for identifying GRN other than OLS? For example, random forest and XGBoost.

5. While the authors have mentioned one of the potential applications of VI-VS is to predict chromatin accessibility in ATAC-seq data (Page 3, Line 29), the real data does not contain a case study with ATAC-seq data. Given scATAC-seq is much sparser than gene expression and protein abundance data, I am wondering if there is limitation of VI-VS applied to scATAC-seq/Multiome data to identify gene regulatory relationship.

6. One of the advantages of VI-VS compared to marginal is when there is a high correlation between features of X. It will be good if the authors can demonstrate via simulation how VI-VS performs when the correlation of features increases.

**Reviewer 2**

This study introduces a framework, VI-VS, for identifying conditionally dependent features in single-cell omics data while controlling the false discovery rate. VI-VS provides a general wrapper that enables the execution of the conditional randomization test (CRT) on various statistical and machine learning models describing feature relationships. The framework is statistically rigorous. Although CRT and its underlying theory are not entirely new, their adaptation to single-cell omics applications is novel and potentially highly useful. Thus, this work should be documented in the literature. Overall, the manuscript is well-written and easy to follow. Below are several issues I recommend the authors address to further improve the quality of the paper.

Major:

1. Additional benchmarking using real data is needed to more convincingly demonstrate VI-VS's ability to identify potential causal relationships. Currently, the benchmark heavily relies on semi-synthetic data. In the two real data examples (CITE-seq and spatial transcriptomics), the identified feature relationships are compared to existing knowledge. However, since this knowledge is incomplete, the benchmark is not very systematic. For instance, simple differential expression tests detected almost all genes as features associated with tumor and lymphoid aggregates. This only "suggests" that these tests may report too many false discoveries, but it does not provide objective proof based on ground truth, which may not be readily available. As the authors mentioned, Perturb-seq offers a systematic way to validate causal candidates identified by VI-VS and other methods. With many Perturb-seq datasets available, benchmarking VI-VS using Perturb-seq would be more systematic and convincing.

2. This study uses 7:3 development-validation split. Would the split ratio influence the power of detecting conditionally dependent features? If yes, is there a way that can inform users how to optimally split the data?

3. The study demonstrated how increasing prediction model complexity influences the detection power (e.g., Fig. 4B,C). A natural question is how increasing the latent variable model (e.g. VAE) complexity would influence the detection power. Similarly, how will different methods/models for generating synthetic samples influence the detection power?

Minor:

4. Formula (7): T(y,x,s) should be changed to T(x,y,s) to be consistent with the notations used elsewhere, where x always precedes y in T(.).

5. Page 6, last sentence of the section "Cell-specific scores": "positive scores will highlight that randomizing the gene g in cell x [decreases] the predictive loss." Shouldn't positive scores reflect an [increase] of loss rather than a [decrease]?

**Authors’ response**

Editor

As you will see from the reports, both referees are broadly favorable and find the work of potential interest, but they raise important issues that we must ask you to address, in the form of a revised manuscript, before we reach a final decision on publication. **In particular, both referees ask that you show how robust the method is to different data splits**. **Referee 1 asks that you demonstrate the method with different levels of correlation**. **Referee 2 asks that you validate the method using Perturb-seq data.** In addition, Referee 1 asks that you demonstrate an application using ATAC-seq data. We think this would strengthen the manuscript, and we would encourage you to do it, but we will not insist on it. You should, though, **discuss how the increased sparsity of ATAC-seq data would affect the method**. Please ensure that these and all other issues raised by the referees are addressed in full.

We have addressed these three major comments, as detailed below (other issues are answered on a per-comment basis in our response to the referees):

**1. Robustness to data splits.** We added an additional experiment in our semi-synthetic assay, where we measured VI-VS calibration and power for different development/validation ratios. We observed stable and robust behaviors for VI-VS across ratios where the development ratios were within the range of common practice (i.e., higher than 50%; **Supplement Information D.5**). We also used observations from this experiment to suggest a new way to identify the best development/validation split for a given dataset, based on the evidence lower bound (ELBO) curve.

**2. Performance of VI-VS with different levels of correlation.** We devised a semi-synthetic experiment where we had knowledge of ground-truth conditional associations, and where the correlations between pairs of features could be set (**Supplement Information D.1**). This experiment provided a richer comparison of conditional and marginal independence tests and also supported the notion of making inferences both at the gene and module level. Specifically, we show that true positive genes that could not be identified at the gene level (due to strong correlations) could be identified at our coarser (module- level) analysis, illustrating the relevance of the multi-resolution approach.

**3. Validation using perturb-seq.** We added a Perturb-seq experiment to our manuscript in **Section 2.3**. We used both observational and perturbational data to identify ground-truth gene-gene associations for the ten targeted transcription factors (TFs). Comparing VI-VS to the OLS and marginal baselines, we find that it produced the most precise detections with comparable recall than OLS.

**4. Application to ATAC-seq data.** We believe the identification of chromatin accessibility to gene expression to be a promising application of our method. In particular, we believe the multi-resolution component of VI-VS to be particularly relevant for the identification of potential transcription factor binding sites without the need to settle on a specific resolution. Unfortunately, evaluating VI-VS for scATAC + scRNA-seq data is challenging due to the absence of ground-truth. However, we now refer to this point in the discussion section, and highlight the generative modeling schemes, by us (Ashuach et al. 2023, 2022) and others (Martens et al. 2024), that can be harnessed for this purpose.

Reviewer 1:

Boyeau et al. introduces a new framework, VI-VS, to explore conditionally dependent features using variational inference. VI-VS quantifies the credibility of pairwise interactions of the observed features compared to the synthetic ones, without making assumptions about the distribution of the response variables and is able to capture the non-linear relationship between variables using a neural network framework. Overall, this paper proposed a very interesting formulation and framework to identify feature dependencies in single-cell datasets.

I have the following comments of the manuscript.

1. The methods are based on the previous works of ModelX knockoffs. I am wondering how well using generative model scVI preserve the dependencies between features. How would other ways to construct synthetic gene expression matrix perform compared to scVI, such as scDesign3, scGAN and ZINB-WaVE?

We thank the reviewer for bringing this point up. VI-VS identifies conditional dependencies by using a generative model to produce synthetic data. Within that, VI-VS focuses specifically on latent variable models, following the general form in Equation (3). Many models different from scVI adhere to this form. Variational autoencoders, that have been extensively applied in single-cell genomics (e.g., (Svensson et al. 2020; Martens et al. 2024; Ashuach et al. 2022; Gayoso et al. 2021; Ding and Regev 2021)), constitute a first class of generative models that VI-VS can use. VI-VS can also use simpler latent variable models for which the posterior distribution is tractable (Pierson and Yau 2015).

The models mentioned by the reviewer cannot easily be used as generative models in VI-VS, either because they do not factorize as in Equation (3). To still make the point that VI-VS is not tied to scVI, we considered an alternative latent variable model, ldVAE (Svensson et al. 2020) in the semi-synthetic experiment (**Supplement Information D.4**). Since this model posits a linear relationship between the latent variable and gene expression, it arguably operates on similar assumptions as ZINB-Wave. We observed that ldVAE controlled the false discovery rate, showing that alternative generative models can be employed within VI-VS.

2. How stable is the output of VI-VS for different splits of datasets?

a. We added a simulation experiment to evaluate the impact of the development-validation split ratio on the stability of the predictions. We found that the FDR and power levels were satisfactory and stable across a broad range of ratios, provided that the majority (>50%) of the data is used for training (**Supplement Information D.5**). This comment also prompted us to define an automated way to determine the desirable ratio. In the experiment above, we noticed that the validation negative evidence lower-bound (ELBO) provides a good way to determine the minimally required development-validation ratio. Specifically, if we plot the validation ELBO as a function of the train/validation ratio, we can identify a point in which the model's fit stabilizes and shows minimal improvement with additional data. This point corresponds well to the minimal development-validation ratio that is needed to ensure calibration. This observation allowed us to provide additional guidelines for selecting development-validation ratios for VI-VS, that can also be found in **Supplement Information D.5**.

b. We also added an experiment assessing the stability of discoveries in the CITE-seq experiment, showing a high consistency of discoveries (measured with Jaccard indices) across different development-validation split ratios (**Supplement Information D.7**).

3. Following the last point, how reproducible the results of VI-VS when applying to different batches of data? For example, if there are two technical replicates of spatial data from or different batches of scRNA-seq data, can VI-VS identify similar features?

The inclusion of scVI in VI-VS provides a principled approach to identify batch-independent discoveries, by conditioning, in addition to other gene expressions, on the batch (Luecken et al. 2022; Boyeau et al. 2023). We now added a more detailed discussion on this issue in the discussion section. In addition to mentioning the above, we also refer to a paper by (Barry et al. 2021) that demonstrated the ability to overcome spurious associations caused by technical factors using CRT and a non-parametric approach for modeling these effects.

4. How would VI-VS compare to other non-linear machine learning methods that are commonly used for identifying GRN other than OLS? For example, random forest and XGBoost.

We thank the reviewer for making this important point. A major part of our motivation for developing VI-VS is not as a replacement to such methods, but rather as a wrapper algorithm that enables a principled and interpretable way to identify conditional dependencies (i.e., estimating FDR). In this regard, we refer to **Figure 3E**, where we used VI-VS as a meta-method that can translate random forest predictions into interpretable discoveries. We now emphasize this point further in the Discussion section.

5. While the authors have mentioned one of the potential applications of VI-VS is to predict chromatin accessibility in ATAC-seq data (Page 3, Line 29), the real data does not contain a case study with ATAC-seq data. Given scATAC-seq is much sparser than gene expression and protein abundance data, I am wondering if there is limitation of VI-VS applied to scATAC-seq/Multiome data to identify gene regulatory relationship.

We believe the identification of chromatin accessibility to gene expression to be a promising application of our method. Unfortunately, evaluating VI-VS for scATAC + scRNA-seq data is challenging due to the absence of comprehensive ground-truth. However, we now refer to this point in the discussion section, and highlight the generative modeling schemes (by us (Ashuach et al. 2022, 2023) and others (Martens et al. 2024)) that can be harnessed for this purpose.

6. One of the advantages of VI-VS compared to marginal is when there is a high correlation between features of X. It will be good if the authors can demonstrate via simulation how VI-VS performs when the correlation of features increases.

We thank the reviewer for this question. In the presence of mild correlations among features, conditional approaches like VI-VS can filter spurious features whose relationship with the response can be explained from the other features. This is illustrated, for example, in **Figure 3A**, or in **Table S2**. However, conditional approaches may fail to detect the correlated features (low sensitivity) when correlation is high.

1. To illustrate this point, we conducted an experiment as described by the reviewer. Starting from the same scRNA-seq dataset used in Section 2.2, we devised a semi-synthetic dataset allowing us to control feature correlations. In particular, starting from a random gene ��, we generated a correlated profile for a synthetic gene ��' as �� , where

_��'_ = ��_��_+ ϵ

ϵ ∼ ��������������(λ) is an independent Poisson noise. In particular, as λ decreases, genes �� and ��’ get more correlated. The response �� was synthetically constructed as a function of �� but not ��’, using the procedure described in **Supplement Information C.2**.

2. The results of this experiment can be found in **Supplement Information D.1.** As expected, we observed that as correlation between the genes increased, the gene level adjusted p values for conditional association increased, making it difficult to detect the correct gene ��.

3. This experiment, however, allowed us to illustrate the benefit of multi-resolution approaches since at the group level, the module containing �� was called significant. This motivates multi-resolution hypothesis testing as a tradeoff between the sensitivity and specificity of marginal (limited specificity) and conditional tests (potentially limited sensitivity), and its relevance for single-cell omics across modalities.

Reviewer #2:

This study introduces a framework, VI-VS, for identifying conditionally dependent features in single-cell omics data while controlling the false discovery rate. VI-VS provides a general wrapper that enables the execution of the conditional randomization test (CRT) on various statistical and machine learning models describing feature relationships. The framework is statistically rigorous. Although CRT and its underlying theory are not entirely new, their adaptation to single-cell omics applications is novel and potentially highly useful. Thus, this work should be documented in the literature. Overall, the manuscript is well-written and easy to follow. Below are several issues I recommend the authors address to further improve the quality of the paper.

Major:

1. Additional benchmarking using real data is needed to more convincingly demonstrate VI-VS's ability to identify potential causal relationships. Currently, the benchmark heavily relies on semi-synthetic data. In the two real data examples (CITE-seq and spatial transcriptomics), the identified feature relationships are compared to existing knowledge. However, since this knowledge is incomplete, the benchmark is not very systematic. For instance, simple differential expression tests detected almost all genes as features associated with tumor and lymphoid aggregates. This only "suggests" that these tests may report too many false discoveries, but it does not provide objective proof based on ground truth, which may not be readily available. As the authors mentioned, Perturb-seq offers a systematic way to validate causal candidates identified by VI-VS and other methods. With many Perturb-seq datasets available, benchmarking VI-VS using Perturb-seq would be more systematic and convincing.

We thank the reviewer for bringing up this important point. We added a benchmark experiment based on the perturbational assay from (Frangieh et al. 2021). We used the perturbation results to identify genes that are causally related to the ten targeted transcription factors (TFs; using differential expression test - perturbed vs. control) with the highest number of guides. We then compared this “ground truth” set to the set of genes identified for each TF (used as responses) by VI-VS, as well as by OLS and marginal tests.

Results for this experiment are included in the main text in **Section 2.3** and **Supplement Information D.6** of the revised manuscript, showing that VI-VS compares favorably to both OLS and Marginal. Briefly, VI-VS produced much more precise detections than OLS (almost one thirds of VI-VS discoveries were true positives, compared to 5% with OLS) for comparable recall levels.

2. This study uses a 7:3 development-validation split. Would the split ratio influence the power of detecting conditionally dependent features? If yes, is there a way that can inform users how to optimally split the data?

a. We added a simulation experiment to evaluate the impact of the development-validation split ratio on the stability of the predictions. We found that the FDR and power levels were satisfactory and stable across a broad range of ratios, provided that the majority (>50%) of the data is used for training (**Supplement Information D.5**).

This comment also prompted us to define an automated way to determine the desirable ratio. In the experiment above, we noticed that the validation negative evidence lower-bound (ELBO) provides a good way to determine the minimally required development-validation ratio. Specifically, if we plot the validation ELBO as a function of the train/validation ratio, we can identify a point in which the model's fit stabilizes and shows minimal improvement with additional data. This point corresponds well to the minimal development-validation ratio that is needed to ensure calibration. This observation allowed us to provide additional guidelines for selecting development-validation ratios for VI-VS, that can also be found in **Supplement Information D.5**.

b. We also added an experiment assessing the stability of discoveries in the CITE-seq experiment, showing a high consistency of discoveries (measured with the Jaccard indices) across different development-validation split ratios (**Supplement Information D.7**).

3. The study demonstrated how increasing prediction model complexity influences the detection power (e.g., Fig. 4B,C). A natural question is how increasing the latent variable model (e.g. VAE) complexity would influence the detection power. Similarly, how will different methods/models for generating synthetic samples influence the detection power?

We thank the reviewer for this question, which gives us an opportunity to clarify how importance score and generative model complexity affect VI-VS inferences. To investigate this point, we compared two latent variable model choices, scVI (which uses a complex generative model based on multilayer perceptrons), to ldVAE (which uses a linear generative model; **Table S1**). As expected based on the discussion above, the latent variable model choice did not affect power. However, we also found that it did not affect calibration in this experiment. This observation, taken in addition to experiments based on importance score complexity in the main text (**Figures 3B and 3C**), suggest that VI-VS is robust. However, generally we agree with the reviewer that importance score complexity is more likely to influence power and less so calibration (and vice versa for the LVM complexity). We now discuss this in the Discussion..

Minor:

4. Formula (7): T(y,x,s) should be changed to T(x,y,s) to be consistent with the notations used elsewhere, where x always precedes y in T(.).

We thank the reviewer for identifying the typo; the equation has been corrected.

5. Page 6, last sentence of the section "Cell-specific scores": "positive scores will highlight that randomizing the gene g in cell x [decreases] the predictive loss." Shouldn't positive scores reflect an [increase] of loss rather than a [decrease]?

We clarified this part of the text in the main text.

Bibliography:

Ashuach, Tal, Mariano I. Gabitto, Rohan V. Koodli, Giuseppe-Antonio Saldi, Michael I. Jordan, and Nir Yosef. 2023. “MultiVI: Deep Generative Model for the Integration of Multimodal Data.” *Nature Methods* 20 (8): 1222–31.

Ashuach, Tal, Daniel A. Reidenbach, Adam Gayoso, and Nir Yosef. 2022. “PeakVI: A Deep Generative Model for Single-Cell Chromatin Accessibility Analysis.” *Cell Reports Methods* 2 (3): 100182. Barry, Timothy, Xuran Wang, John A. Morris, Kathryn Roeder, and Eugene Katsevich. 2021. “SCEPTRE Improves Calibration and Sensitivity in Single-Cell CRISPR Screen Analysis.” *Genome Biology* 22 (1): 344.

Boyeau, Pierre, Jeffrey Regier, Adam Gayoso, Michael I. Jordan, Romain Lopez, and Nir Yosef. 2023. “An Empirical Bayes Method for Differential Expression Analysis of Single Cells with Deep Generative Models.” *Proceedings of the National Academy of Sciences of the United States of America* 120 (21): e2209124120.

Ding, Jiarui, and Aviv Regev. 2021. “Deep Generative Model Embedding of Single-Cell RNA-Seq Profiles on Hyperspheres and Hyperbolic Spaces.” *Nature Communications* 12 (1): 2554. Frangieh, Chris J., Johannes C. Melms, Pratiksha I. Thakore, Kathryn R. Geiger-Schuller, Patricia Ho, Adrienne M. Luoma, Brian Cleary, et al. 2021. “Multimodal Pooled Perturb-CITE-Seq Screens in Patient Models Define Mechanisms of Cancer Immune Evasion.” *Nature Genetics* 53 (3): 332–41. Gayoso, Adam, Zoë Steier, Romain Lopez, Jeffrey Regier, Kristopher L. Nazor, Aaron Streets, and Nir Yosef. 2021. “Joint Probabilistic Modeling of Single-Cell Multi-Omic Data with totalVI.” *Nature Methods* 18 (3): 272–82.

Luecken, Malte D., M. Büttner, K. Chaichoompu, A. Danese, M. Interlandi, M. F. Mueller, D. C. Strobl, et al. 2022. “Benchmarking Atlas-Level Data Integration in Single-Cell Genomics.” *Nature Methods* 19 (1): 41–50.

Martens, Laura D., David S. Fischer, Vicente A. Yépez, Fabian J. Theis, and Julien Gagneur. 2024. “Modeling Fragment Counts Improves Single-Cell ATAC-Seq Analysis.” *Nature Methods* 21 (1): 28–31.

Pierson, Emma, and Christopher Yau. 2015. “ZIFA: Dimensionality Reduction for Zero-Inflated Single-Cell Gene Expression Analysis.” *Genome Biology* 16 (November):241.

Svensson, Valentine, Adam Gayoso, Nir Yosef, and Lior Pachter. 2020. “Interpretable Factor Models of Single-Cell RNA-Seq via Variational Autoencoders.” *Bioinformatics* 36 (11): 3418–21.

**2^nd^ round**

**Reviewer 1**

The authors have addressed all the comments I raised.

**Reviewer 2**

The authors have adequately addressed all of my previous concerns.
